# Secreted acid sphingomyelinase as a potential gene therapy for limb girdle muscular dystrophy 2B

**DOI:** 10.1172/JCI141295

**Published:** 2022-01-04

**Authors:** Daniel C. Bittel, Sen Chandra Sreetama, Goutam Chandra, Robin Ziegler, Kanneboyina Nagaraju, Jack H. Van der Meulen, Jyoti K. Jaiswal

**Affiliations:** 1 Center for Genetic Medicine Research, Children’s National Hospital, Washington, DC, USA.; 2 Rare and Neurologic Diseases Research, Sanofi, Framingham, Massachusetts, USA.; 3 School of Pharmacy and Pharmaceutical Sciences, SUNY Binghamton University, Binghamton, New York, USA.; 4 Department of Genomics and Precision Medicine, George Washington University School of Medicine and Health Sciences, Washington, DC, USA.

**Keywords:** Muscle Biology, Gene therapy

## Abstract

Efficient sarcolemmal repair is required for muscle cell survival, with deficits in this process leading to muscle degeneration. Lack of the sarcolemmal protein dysferlin impairs sarcolemmal repair by reducing secretion of the enzyme acid sphingomyelinase (ASM), and causes limb girdle muscular dystrophy 2B (LGMD2B). The large size of the dysferlin gene poses a challenge for LGMD2B gene therapy efforts aimed at restoring dysferlin expression in skeletal muscle fibers. Here, we present an alternative gene therapy approach targeting reduced ASM secretion, the consequence of dysferlin deficit. We showed that the bulk endocytic ability is compromised in LGMD2B patient cells, which was addressed by extracellularly treating cells with ASM. Expression of secreted human ASM (hASM) using a liver-specific adeno-associated virus (AAV) vector restored membrane repair capacity of patient cells to healthy levels. A single in vivo dose of hASM-AAV in the LGMD2B mouse model restored myofiber repair capacity, enabling efficient recovery of myofibers from focal or lengthening contraction–induced injury. hASM-AAV treatment was safe, attenuated fibro-fatty muscle degeneration, increased myofiber size, and restored muscle strength, similar to dysferlin gene therapy. These findings elucidate the role of ASM in dysferlin-mediated plasma membrane repair and to our knowledge offer the first non–muscle-targeted gene therapy for LGMD2B.

## Graphical abstract

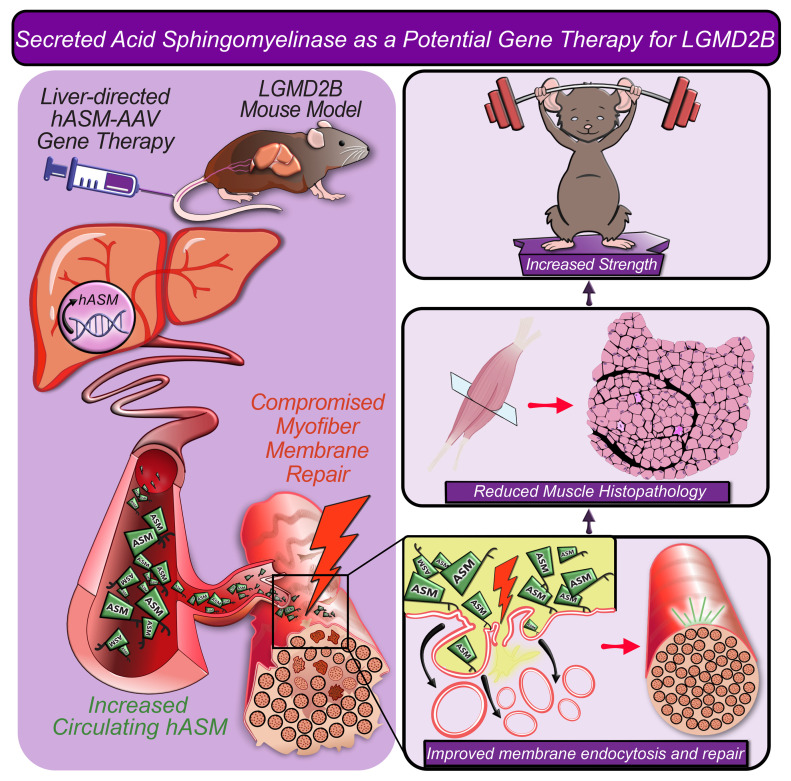

## Introduction

Skeletal muscle cells (myofibers) enable physical movement and are frequently damaged by strenuous activity, overload, and eccentric contractions (1, 2). Mutations that increase myofiber fragility or impede repair result in muscle degeneration and muscular dystrophies (3). Miyoshi myopathy (MM) and limb girdle muscular dystrophy 2B (LGMD2B) are two such autosomal recessive muscular dystrophies that manifest in early adulthood and lead to progressive skeletal muscle weakness and wasting (4). These diseases (collectively called dysferlinopathy) are caused by mutations in the *DYSF* gene, which encodes a large (237 kDa) muscle membrane protein — dysferlin (5, 6). Even prior to overt muscle degeneration, dysferlinopathic patient myofibers exhibit plasma membrane (sarcolemma) defects, including membrane tears, extrusions, subsarcolemmal accumulation of vesicles and vacuoles, and thickening of the basal lamina (7). These early abnormalities are suggested to be caused by poor repair of sarcolemmal injury (7, 8). Damage to the myofiber sarcolemma is repaired by a complex signaling process activated by the injury-triggered influx of extracellular calcium, which is compromised by dysferlin deficit (9, 10). Failed or deficient myofiber repair activates chronic inflammatory responses and leads to muscle degeneration — a notable feature of dysferlinopathic skeletal muscle (11–13).

Repair of plasma membrane injury involves calcium-triggered vesicle shedding and fusion, facilitated by calcium binding proteins that include synaptotagmins (9, 14–22). Similar to synaptotagmins, dysferlin is a member of the C2 domain protein family that bind negatively charged membrane phospholipids in a calcium-dependent manner (23, 24). We have shown that dysferlin mediates sarcolemmal repair by tethering lysosomes to the plasma membrane, facilitating immediate lysosome exocytosis upon membrane injury (10). This allows the lysosomal enzyme acid sphingomyelinase (ASM) to be secreted within seconds of sarcolemmal injury, which is required for repair (25, 26). Lack of dysferlin delays and reduces injury-triggered lysosome exocytosis, thereby slowing and reducing ASM secretion by the injured cell (10). Consequentially, reduced ASM secretion by the injured dysferlinopathic muscle cells or lack of ASM production in Niemann-Pick disease type A (NPDA) cells compromises their sarcolemmal repair (10, 26). These deficits identify extracellular ASM supplementation as a potential treatment to improve myofiber repair for both LGMD2B and NPDA patients.

Upon secretion into the extracellular medium, ASM hydrolyzes the plasma membrane sphingomyelin lipids to ceramide, to help remove damaged portions of the plasma membrane through extracellular vesicle (ECV) shedding and by endocytosis (25, 27). Plasma membrane injured by pore-forming toxins has been found to undergo both ECV shedding and caveolar endocytosis (28, 29). The toxins colocalize with glycosylphosphatidylinositol (GPI), which marks endosomes formed by clathrin-independent carriers (CLICs) (30, 31). However, details of how ASM helps repair physiological (focal or mechanical) injury to the plasma membrane remain unresolved. Understanding the role of ASM in repair of physiological membrane injury is crucial for informing treatments for diseases involving mechanically induced plasma membrane injuries that affect muscle, lung, and other organs.

Preclinical gene therapy approaches for LGMD2B that involve reexpressing the dysferlin gene in skeletal muscle have resulted in a mixed, but overall positive, therapeutic outlook (32–34). The progress of these therapies to the clinic, however, requires overcoming barriers associated with the efficient packaging and skeletal muscle delivery of the large dysferlin gene (35). Drug-based therapies offer an alternative, but there are currently no approved drugs to address this or other disease etiology of dysferlinopathy. Preclinical studies indicate that drugs that stabilize the sarcolemma can enable myofiber repair and improve dysferlinopathic muscle function (36, 37). Our previous studies show that ASM improves dysferlinopathic myofiber repair (10). Preclinical and human studies using intravenous delivery of hASM and liver-targeted hASM delivered via adeno-associated virus (hASM-AAV) have shown their efficacy for other indications (38–41). Recent work has also demonstrated clinical safety of hASM for treating NPDA (42, 43). However, the utility of this approach for improving skeletal muscle deficits in LGMD2B or NPDA has not been tested. Here, we examine the in vitro efficacy of hASM protein and in vivo efficacy of non–muscle-targeted hASM-AAV gene therapy to improve sarcolemmal repair using patient muscle cells and LGMD2B mouse models. We also leverage the mouse model for LGMD2B to examine the potential of hASM-AAV gene therapy for chronic improvement of myofiber repair, muscle histopathology, and muscle function.

## Results

### hASM restores patient cell repair independently of vesicle shedding and caveolar endocytosis.

**Figure 1:**
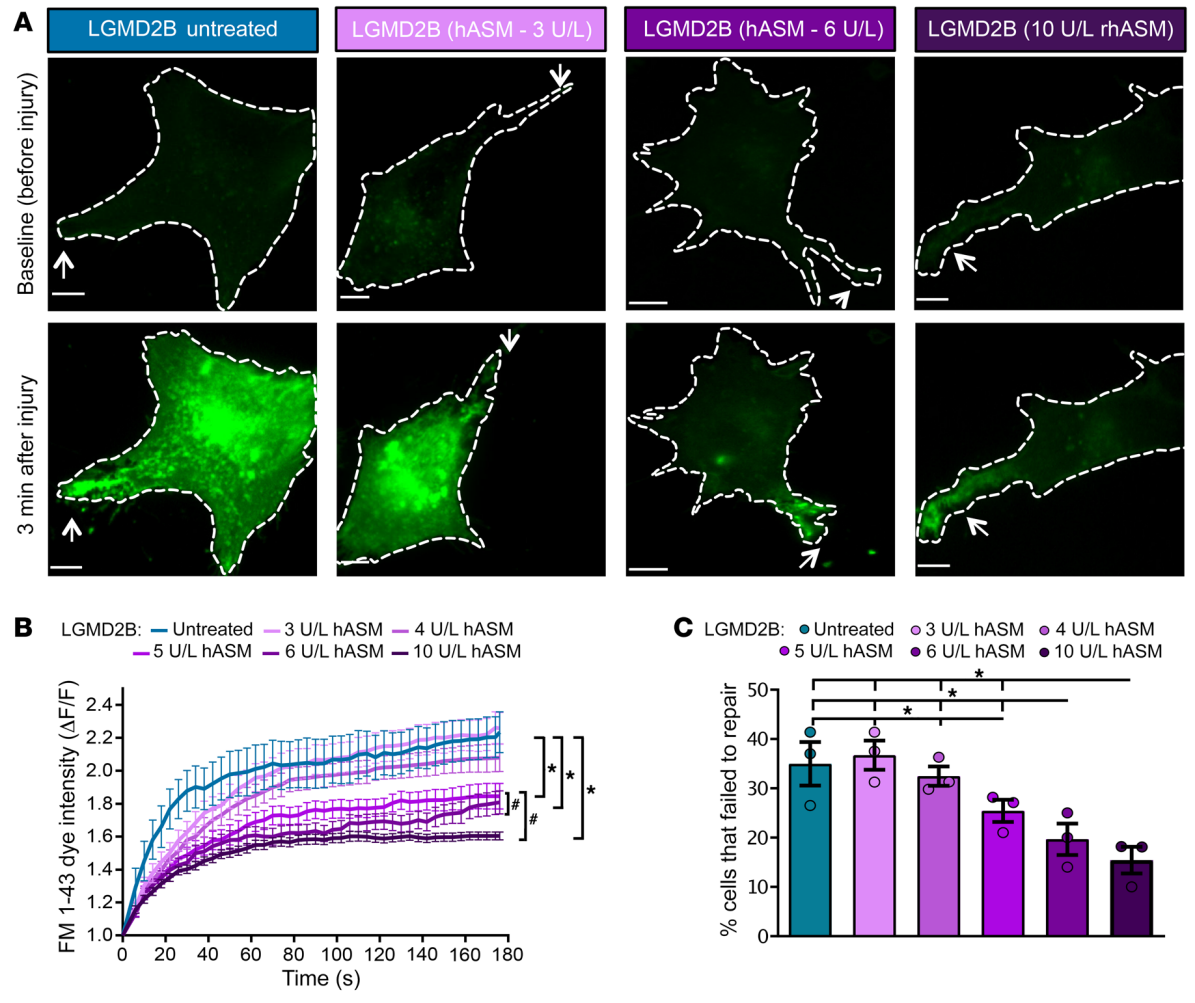
hASM improves LGMD2B patient cell repair in a dose-dependent manner. LGMD2B patient myoblasts were treated with increasing doses of purified hASM protein. (**A**) Confocal images of the myoblasts prior to and following focal laser injury (site marked by white arrow) showing FM 1-43 dye (green) labeling. (**B**) Plot showing the kinetics of FM dye entry into myoblasts following membrane injury (*n =* 50 cells per condition). *P < 0.05 (vs. untreated and 3 U/L); ^#^*P <* 0.05 (vs. 5 U/L) by mixed-model ANOVA with analyses for interaction effects between treatment condition and time. (**C**) Quantification of the proportion of laser-injured cells that fail to repair (*n* > 45 cells per condition). One-way ANOVA with Tukey’s HSD post hoc test, with α set at *P <* 0.05 (*n =* 3 experimental repeats with 15–18 cells per repeat per condition). Data are presented as mean ± SEM. Scale bars: 10 μm.

To test the effect of hASM on plasma membrane repair, primary human myoblasts from LGMD2B patients were treated with purified hASM protein. Exposure to purified hASM led to a dose-dependent improvement in patient cell membrane repair (Figure 1, A–C). Purified hASM at a concentration of 3 U/L or 4 U/L was not efficacious in improving repair of patient myoblasts (Figure 1, A and B). However, treating the patient cells with purified-hASM doses over 4 U/L significantly improved plasma membrane repair, reducing FM 1-43 dye entry into the injured cells (Figure 1, A and B). A clear dose-dependent effect of hASM on patient cell membrane repair emerged, such that 5 U/L hASM improved membrane repair, and the effect peaked at concentrations of 6 and 10 U/L hASM (Figure 1, B and C). Consequently, while 5 U/L hASM reduced the number of cells that failed to repair, greatest improvement was attained at the hASM dose of 6 U/L and higher (Figure 1C).

**Figure 2:**
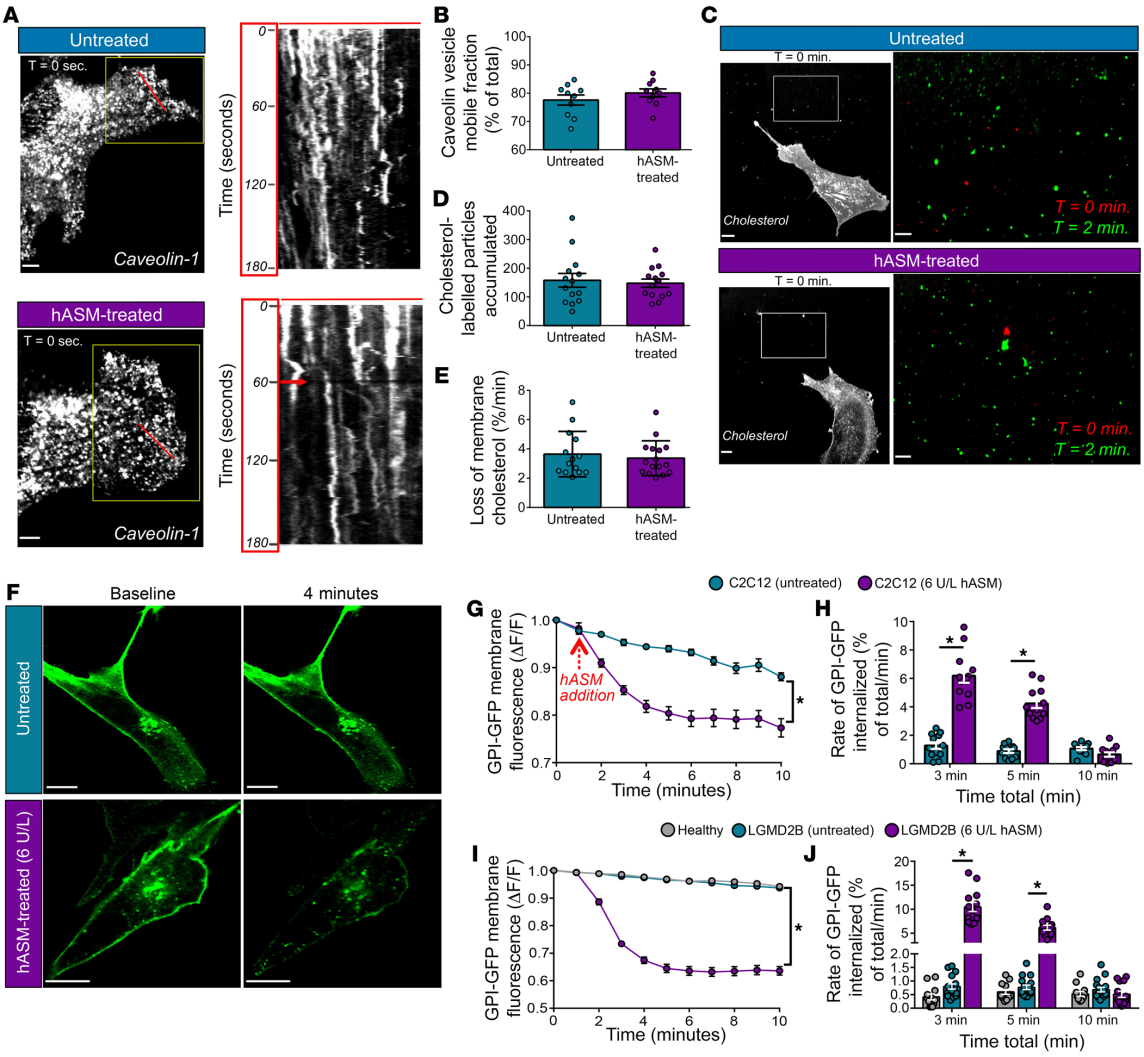
hASM activates endocytosis via the CLIC/GEEC pathway. (**A**) Confocal images of the bottom surface (cell-coverslip interface) of mouse myoblasts expressing mRFP-tagged caveolin-1 either untreated (top) or treated with 6 U/L purified hASM (bottom). Grayscale image shows the whole cell at the start of imaging (time point 0). The red line on the cell marks the pixels shown in the grayscale kymograph demonstrating caveolin-1 mobility, presented as images acquired at 1 frame per second, for a 3-minute period (kymograph *y* axis = total acquisition time of 180 seconds). Note the broken tracks of pixels, indicating movement of caveolae present at the cell membrane. The red arrow in the hASM-treated kymograph indicates the time of hASM addition at the 60-second mark. (**B**) Plot showing quantification of caveolin-1 puncta in each condition (*n =* 50 puncta from 10 cells). (**C**) To track cell membrane shedding, live cells were labeled with FITC-PEG-cholesterol prior to imaging. Grayscale images show confocal image of the cell membrane at the coverslip surface at the start of imaging (time point 0), and the white box marks the extracellular space on the coverslip adjacent to the cell used to monitor the cholesterol-labeled vesicles shed by the cell. The zoom of this region is shown in the pseudocolored panels on the right, where red color indicates vesicles present at the onset of imaging (baseline), and green color indicates vesicles present 2 minutes after mock (untreated) treatment or treatment with 6 U/L hASM (hASM-treated). (**D**) Quantification of FITC-PEG-cholesterol–enriched particles shed by cells treated or not treated with 6 U/L hASM (*n =* 10 cells per condition). (**E**) Quantification of the rate of loss of cell-associated FITC-PEG-cholesterol fluorescence by the cells imaged in **C** and **D** (*n =* 10 cells per condition). (**F**) Images showing an optical section through the middle of mouse myoblasts expressing the CLIC/GEEC reporter GPI-GFP before and 4 minutes after treatment with 6 U/L hASM. (**G**–**J**) Plots showing (**G** and **I**) kinetics and (**H** and **J**) rate of internalization of GPI-GFP in (**G** and **H**) C2C12 myoblasts and (**I** and **J**) healthy and patient myoblasts. Data represent mean ± SEM. *P < 0.05 (vs. untreated cells) via independent samples *t* test (**B**, **D**, and **E**); kinetics and rate-analyses were performed via mixed-model ANOVA, with α set at *P <* 0.05 (**G**–**J**). Scale bars: 10 μm and 5 μm (zoomed images).

With the involvement of caveolar endocytosis and membrane shedding in repairing membrane injury by pore-forming toxins, we examined the effect of hASM on these pathways. We monitored caveolar endocytosis by live imaging of caveolae dynamics in myoblasts expressing caveolin-1 tagged with monomeric red fluorescent protein (mRFP) (Supplemental Figure 1A and Supplemental Figure 2A; supplemental material available online with this article; https://doi.org/10.1172/JCI141295DS1). Imaging individual plasma membrane–associated caveolae by confocal microscopy showed that within a 2-minute period over 75% of caveolae at the plasma membrane moved from their starting position, and treatment with 6 U/L purified hASM did not affect this fraction of mobile caveolae (78% ± 1.8% vs. 80% ± 1.4%; Figure 2, A and B). Next, we examined whether this dose of hASM triggers plasma membrane shedding. As ECVs are enriched in cholesterol (20), we labeled the cell membrane with FITC-PEG-cholesterol and quantified vesicle shedding over a 2-minute period (Supplemental Figure 1B and Supplemental Figure 2C). Untreated and 6 U/L hASM–treated cells shed similar numbers of ECVs — untreated, 158 ± 24; hASM-treated, 147 ± 15 (Figure 2D) — and led to the loss of similar amounts of cell-associated cholesterol labeling (Figure 2E). These results determined that the dose of hASM that improves membrane repair in patient cells did not enhance caveolar endocytosis or ECV shedding, alluding to alternative mechanisms by which hASM improves membrane repair.

### hASM treatment enhances bulk plasma membrane endocytosis.

As a cell’s bulk endocytosis is supported by CLICs, and CLICs facilitate endocytosis of dysferlin and pore-forming toxins (30, 44), we next examined the effect of hASM treatment on GPI-GFP protein, which is used to mark CLICs (Supplemental Figure 1C). Using C2C12 myoblasts, we observed a steady endocytosis of CLICs from the plasma membrane, which was acutely enhanced by treatment with hASM (Figure 2, F–H). We observed a similar rate of CLIC endocytosis in untreated healthy and LGMD2B patient myoblasts (Figure 2, F, I, and J). Similar to the increase in CLIC endocytic rate in mouse myoblasts (Figure 2H), hASM treatment of patient myoblasts also increased the CLIC endocytic rate (Figure 2, I and J).

**Figure 3:**
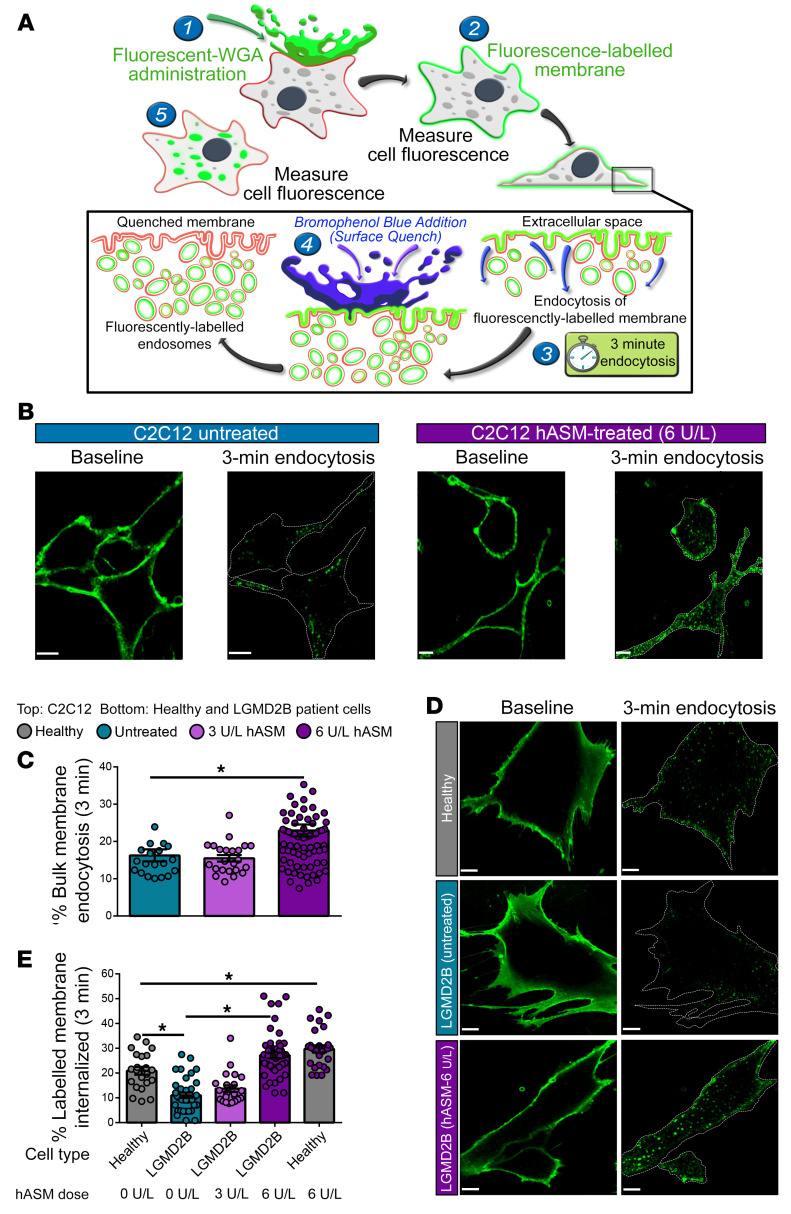
hASM rescues bulk endocytosis deficit in the LGMD2B patient cells. (**A**) Schematic showing the assay used to monitor bulk endocytosis. (1 and 2) The plasma membrane was labeled with fluorescent WGA, and (3) membrane endocytosis was monitored over a 3-minute period by (4) quenching the WGA fluorescence at the cell surface by using bromophenol blue (BPB) at the end of endocytosis period. Punctate fluorescence in the cell, not quenched by BPB, marks the internalized WGA localized in endosomes. (5) Internalized WGA fluorescence was expressed relative to the baseline labeling prior to quenching. (**B**) Confocal image of mouse myoblast labeled with WGA at baseline and after 3 minutes of endocytosis in untreated and hASM-treated cells. (**C**) Plot showing the effect of different doses of hASM on bulk membrane endocytosis in mouse myoblasts. (**D**) Confocal images showing fluorescent WGA–labeled healthy and patient myoblasts at baseline (left panel) and after 3 minutes of endocytosis (right panel). (**E**) Plot showing quantification of bulk endocytosis by healthy and LGMD2B patient muscle cells and the effect of hASM on patient cell endocytosis (*n* > 2 experimental repeats per condition). All data are presented as mean ± SEM. *P < 0.05 (vs. untreated cells), assessed via 1-way ANOVA with Tukey’s HSD post hoc test (**C** and **E**). Scale bars: 10 μm.

With the role of CLICs in bulk membrane removal, we next examined the role of bulk membrane endocytosis in repair by using the lectin wheat germ agglutinin (WGA) to label the plasma membrane and assess its endocytic removal in response to different doses of hASM (Figure 3A). Untreated mouse myoblasts and those treated with 3 U/L hASM endocytosed similar amounts of the plasma membrane–associated WGA, but treatment with 6 U/L hASM significantly increased the rate of WGA endocytosis in mouse muscle cells (untreated, 16.2% ± 1.6%; 3 U/L hASM, 15.5% ± 0.9%; 6 U/L hASM, 23% ± 1.6% WGA internalized) (Figure 3, B and C).

In accordance with our previous findings of reduced ASM secretion by LGMD2B patient myoblasts (10), these cells exhibited 2-fold reduction in their ability to endocytose WGA (11% ± 1% patient vs. 21% ± 1.6% healthy) (Figure 3, D and E). Treatment with the hASM dose that improved LGMD2B cell repair (6 U/L) also enhanced WGA endocytosis of these cells, while the lower dose (3 U/L) failed to do so (Figure 3, D and E). Treating healthy muscle cells with 6 U/L hASM also increased WGA endocytosis (untreated, 20.9% ± 1.6; 6 U/L hASM, 29.7% ± 1.4%; Figure 3E), without causing any cellular toxicity (Supplemental Figure 5). These findings show that hASM-mediated improvement in repair safely enhances bulk plasma membrane endocytosis.

### hASM-AAV offers a genetic approach to restore membrane repair in LGMD2B.

**Figure 4:**
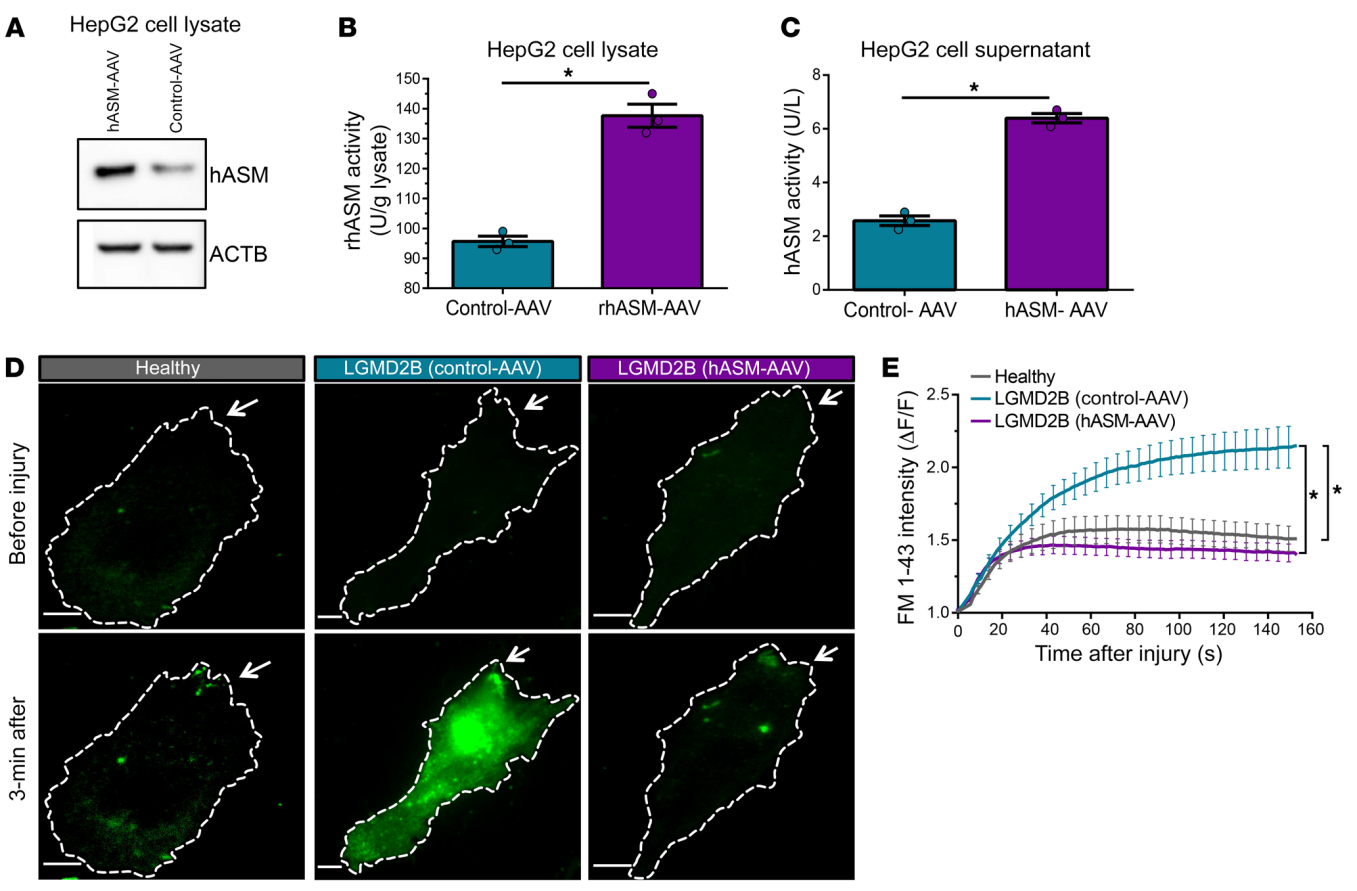
ASM produced by human liver cell–targeted hASM-AAV improves repair of patient myoblasts in culture. (**A**) Western blot for hASM and (**B**) quantification of hASM activity in lysates from HepG2 cells transduced with either control-AAV or hASM-AAV under a liver-specific promoter (*n =* 3 independent replicates). (**C**) Quantification of hASM activity in culture supernatants from control-AAV– and hASM-AAV–infected HepG2 cells (*n =* 3 independent replicates). (**D**) Confocal images of healthy and LGMD2B patient myoblasts prior to and following focal laser injury (site marked by white arrow) showing FM 1-43 dye (green) labeling. LGMD2B patient myoblasts were treated with culture supernatants from control and hASM-AAV–infected HepG2 cells in CIM. (**E**) Plot showing the averaged kinetics of FM-dye entry in healthy and patient myoblasts (*n* > 15 cells per condition). Data are presented as mean ± SEM. *P < 0.001 (vs. control-AAV–treated cells) by independent samples *t* test (**B** and **C**) or mixed-model ANOVA with analyses for interaction effects between treatment condition and time was used (**E**, vs. control-AAV–treated cell supernatant). Scale bars: 10 μm.

While the above studies demonstrate the utility of hASM treatment to safely address the bulk endocytosis defect in the LGMD2B patient cells, for its therapeutic utility the protein will require frequent administration to maintain a therapeutic level in vivo. To overcome this challenge, we explored the use of an alternative approach by genetically expressing secreted hASM to maintain a stable therapeutic level of this protein in the serum. We used an AAV vector to express the secreted form of hASM protein under the control of a liver-specific promoter (hASM-AAV) (45), which we first assessed in vitro by infecting the human liver cell line HepG2. Compared with the control vector, HepG2 cells infected with hASM-AAV secreted 6.4 U/L hASM (Figure 4, A–C). As this is above the therapeutic dose needed to improve membrane repair (6 U/L), we tested the ability of secreted hASM produced by the HepG2 cells to improve the repair of injured LGMD2B patient muscle cells. Compared with the patient myoblasts treated with the culture supernatant from control-AAV–expressing HepG2 cells, patient myoblasts treated with the supernatant of hASM-expressing HepG2 cells repaired efficiently, with kinetics similar to that of the healthy donor myoblasts (Figure 4, D and E). These findings established the in vitro efficacy of liver-targeted hASM-AAV gene therapy to improve plasma membrane repair in LGMD2B patient muscle cells.

### Myofiber sarcolemmal repair is improved by liver-targeted hASM-AAV.

**Figure 5:**
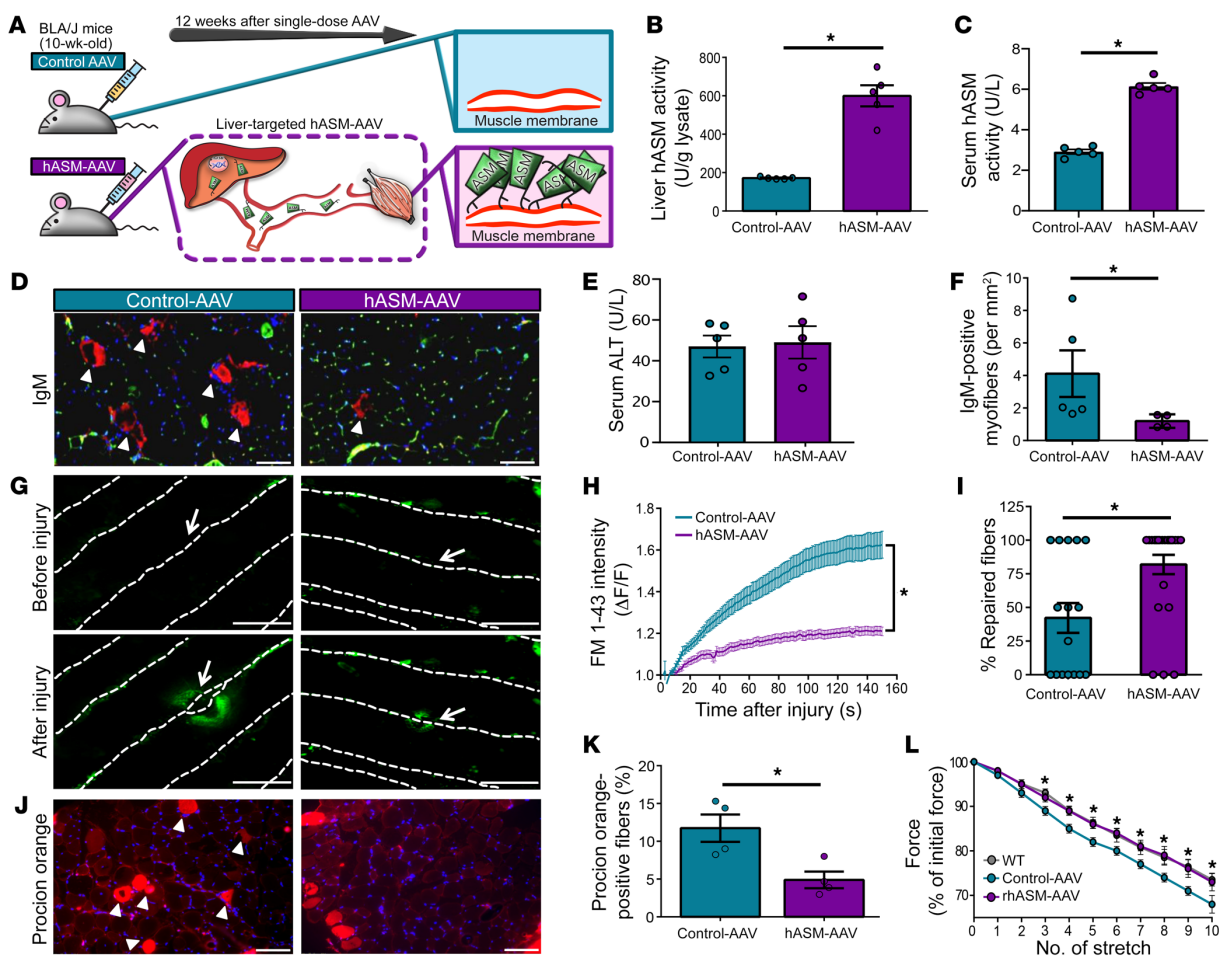
Liver-targeted hASM gene therapy improves dysferlinopathic myofiber repair. (**A**) Schematic showing the gene therapy approach used in vivo in a dysferlin-deficient mouse model for LGMD2B (B6A/J). Twelve weeks after a single i.v. dose of liver-specific hASM-AAV or control-AAV (1.1 × 10^13^ vg/kg), tissues were isolated for functional measurements. (**B**) Plot showing hASM activity in the livers isolated from hASM-AAV– and control-AAV–injected mice (*n =* 5 mice per condition). (**C**) Plot showing hASM activity in the serum of hASM-AAV and control-AAV 12 weeks after injection (expressed in U/L). (**D**) Images of myofibers labeled with anti-IgM antibodies (arrowheads show IgM-positive myofibers). (**E**) Plot showing serum alanine transaminase (ALT) concentration to assess extent of liver damage in control-AAV– and hASM-AAV–treated mice 12 weeks after injection. (**F**) Quantification of IgM-positive myofibers in **D**. (**G**) Images and (**H**) kinetics of FM-dye uptake by myofibers in freshly isolated biceps, following focal laser injury at site marked by white arrow (*n =* 20 myofibers per mouse). (**I**) Plot showing the myofibers that successfully repaired after laser injury (*n* > 15). (**J**–**L**) Isolated EDL muscles from control-AAV– and hASM-AAV–treated mice were injured by repeated 10% eccentric contractions and sarcolemma damage was monitored by labeling with procion orange (PO) dye. (**J**) Images showing the PO-labeled fibers (arrowheads) and (**K**) quantification of the number of PO-labeled fibers per muscle (*n =* 4 muscle per group due to excess damage during preparation of EDL muscle in 1 mouse in each group). (**L**) Change in muscle contractile force with 10 repeated eccentric contractions (*n =* 5 mice per group). Data are presented as mean ± SEM. *P < 0.05 vs. control-AAV. Group differences in tissue hASM activity (**B**), serum hASM activity (**C**), serum ALT activity (**E**), IgM^+^ myofibers (**F**), percentage of repair fibers (**I**), and PO^+^ myofibers (**K**), all assessed via independent samples *t* test. Serum hASM activity (**C**), repair kinetics (**H**), and eccentric force decrement analyses (**L**), were performed via mixed-model ANOVA with analyses for interaction effects between treatment condition and time. Scale bars: 50 μm (**G**) and 100 μm (**D** and **J**).

To test the in vivo efficacy of hASM in improving plasma membrane repair in dysferlin-deficient LGMD2B skeletal muscle fibers, we made use of the dysferlin-deficient B6A/J mouse model of LGMD2B. Signs of muscle damage, myofiber repair deficit, and locomotor deficits are evident in dysferlin-deficient mice by 10 to 24 weeks of age and these progressively worsen, leading to reduced locomotor activity (10, 13, 46–50). By 6 months of age, B6A/J mice show varied muscle histopathology that is pronounced in the quadriceps (specifically the rectus femoris) muscle, while the vastus muscles in the quadriceps and the gastrocnemius muscle are largely spared at this age (13, 47, 48, 51). The rectus femoris muscle undergoes the most extensive myofiber lengthening during gait (among quadriceps muscles) and is the primary knee extensor that prevents knee collapse under body weight during activity (47, 52, 53). These high contractile demands during normal physical activity cause the rectus femoris to be more susceptible to sarcolemmal disruption. Indeed, in the 6-month-old B6A/J mice, we found that compared with the gastrocnemius muscle, quadriceps muscle showed greater degeneration (Supplemental Figure 3, A and B). This degeneration was localized to the rectus femoris muscle, which also showed increased regeneration and inflammation (Supplemental Figure 3, C–E). To assess the effect of hASM-AAV treatment on B6A/J rectus muscle histopathology, mice were treated by a single tail vein injection of liver-specific hASM-AAV or control-AAV at 10 weeks of age followed by 12 weeks of treatment (Figure 5A). Mice treated with hASM-AAV showed a 4-fold higher liver hASM activity and 2-fold higher serum hASM activity as compared with those treated with control-AAV (600 ± 54.7 U/gram vs. 171.6 ± 2.4 U/gram; Figure 5, B and C). This led to an increased serum hASM activity within 1 week of hASM-AAV injection and a sustained higher level of hASM in the serum and muscle of the B6A/J mice treated with hASM-AAV even 12 weeks after treatment (Figure 5C and Supplemental Figure 4). This increased hASM level had no adverse effect on the liver or the overall health of the animal, as assessed by the liver histopathology, serum levels of alanine aminotransferase (ALT), and growth of animals over the 12 weeks of treatment (Figure 5, C and E, and Supplemental Figure 5).

Given the demonstrable improvement imparted by hASM in muscle cell repair in vitro, we assessed whether in vivo hASM treatment improved sarcolemmal repair and reduced myofiber damage and degeneration. Using the presence of IgM as an indicator of myofiber damage, we observed that treatment of mice with hASM-AAV led to a 3-fold reduction in the extent of damaged myofibers in quadriceps muscle (Figure 5, D and F). To directly assess the benefit of the in vivo hASM treatment on myofiber repair, we used an ex vivo laser-induced-injury assay for monitoring myofiber repair in intact biceps muscles from mice treated with control and hASM-AAV. Using this approach, we showed improved repair ability of myofibers from biceps of B6A/J mice treated with hASM-AAV as compared with those treated with control-AAV (Figure 5, G–I). As laser-induced injury is focal, we also examined the repair ability of myofibers injured via mechanical activity. Repair from mechanical activity–induced injuries was assessed in intact extensor digitorum longus (EDL) muscles from hASM-AAV– and control-AAV–treated B6A/J mice and age-matched WT mice. The muscles were injured by 10 bouts of 10% eccentric contractions, which was followed by labeling the injured myofibers using the membrane impermeant vital dye, procion orange (PO). The mechanical injuries resulted in PO labeling of the injured myofibers that failed to repair, and this was reduced by over 2-fold in hASM-AAV–treated mice (Figure 5, J and K). Improved myofiber repair of hASM-AAV–treated mice led to attenuation of muscle force loss due to mechanical injury and was indistinguishable from the healthy (WT) muscle (Figure 5L and Supplemental Figure 6). These findings indicate that chronic in vivo exposure of dysferlinopathic muscle to elevated hASM restores myofiber repair capacity following spontaneous injuries in vivo, as well as focal or mechanical injury ex vivo.

### Preclinical benefits of hASM-AAV for LGMD2B.

**Figure 6:**
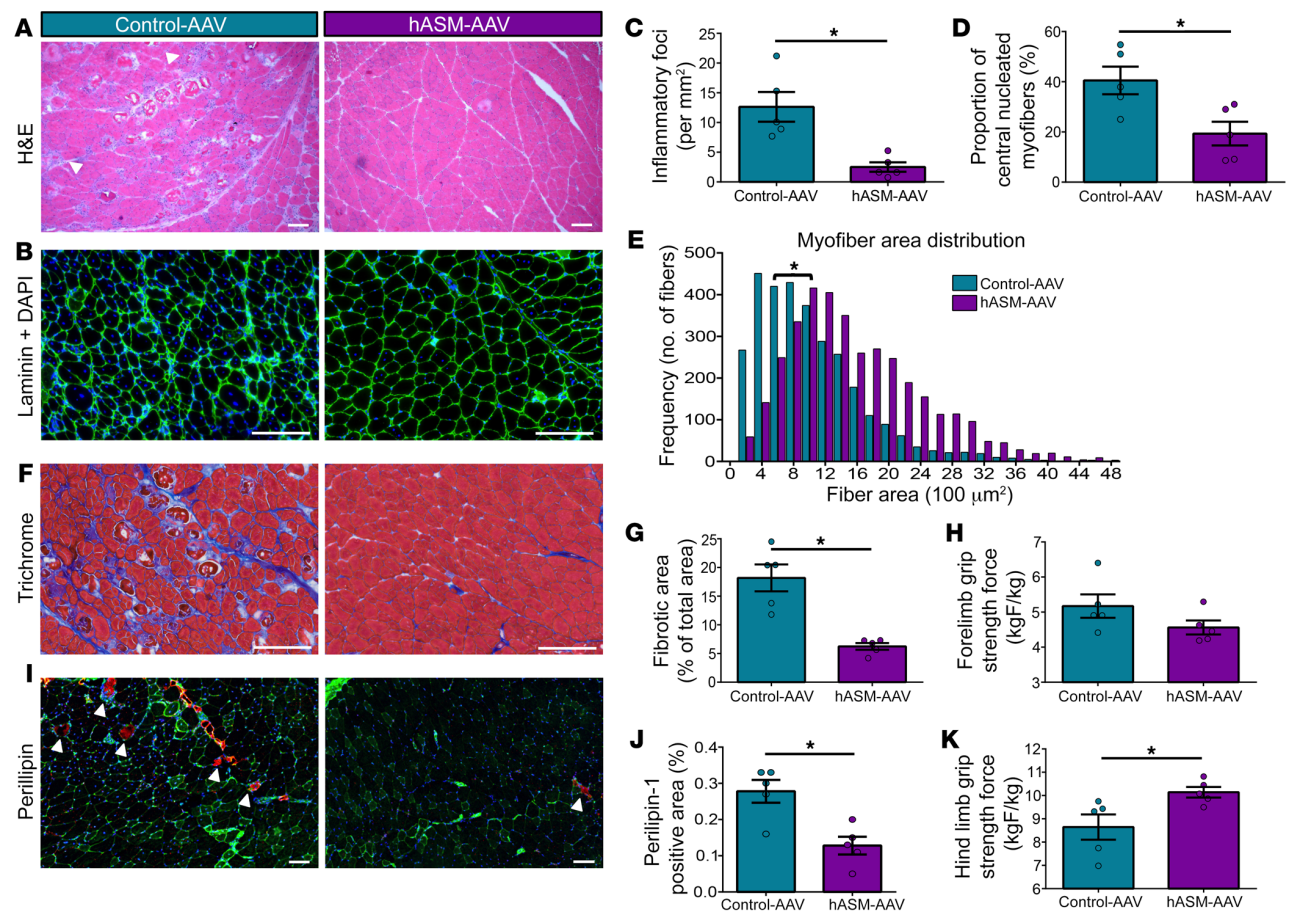
Single dose of liver-targeted hASM-AAV improves muscle histology and function in LGMD2B model. (**A**) H&E-stained image of quadriceps (rectus femoris) muscle cross sections of B6A/J mice after treatment with a single intravenous dose of control-AAV or hASM-AAV (arrowheads mark inflammatory foci). (**B**) Images showing rectus femoris muscle cross sections labeled using anti-laminin antibodies (green) and DAPI (blue) to visualize basement membrane and myonuclei, respectively. (**C**) Plot showing quantification of inflammatory foci in the quadriceps (rectus femoris) muscle cross section similar to those shown in panel **A** (*n =* 5 mice per group). (**D**) Plot showing quantification of centrally nucleated myofibers across the rectus femoris muscle as shown in panel **A**, expressed as percentage of total fibers. (**E**) Distribution of myofiber cross-sectional areas across the entire quadriceps (*n =* 3,000 fibers per group). (**F**) Images and (**G**) quantification of Masson’s trichrome staining of the rectus femoris muscle cross section (*n =* 5 per group). (**H**) Quantification of forelimb grip strength of mice treated as indicated, with the contractile force normalized to body weight (*n =* 5 mice per group, average of 5 repeat measures per mouse. (**I**) Images and (**J**) quantification of perilipin-positive area in the rectus femoris muscle cross section (red, perilipin; green, WGA). (**K**) Quantification of hindlimb grip strength of mice treated as in panel **H**. Data are presented as mean ± SEM. *P < 0.05 (hindlimb) vs. control-AAV (forelimb, *P* > 0.05). Scale bars: 100 μm (**A** and **I**) and 200 μm (**B** and **F**). Data were assessed via independent samples *t* test (**C**, **D**, and **G**–**K**) or Mann-Whitney *U* test (**E**), with α set at *P <* 0.05.

With the above beneficial effects of hASM-AAV therapy for treating the poor myofiber repair and excessive myofiber necrosis caused by dysferlin deficiency, we examined whether this treatment can also improve in vivo muscle histopathology and function. As routine physical activity results in inflammation and degeneration of the quadriceps (rectus femoris) muscle by 6 months of age in the B6A/J mice, we assessed whether hASM-AAV treatment reverses this. Twelve weeks of hASM-AAV treatment of B6A/J mice reduced inflammation (Figure 6, A and C, and Supplemental Figure 7, A and B). Given that chronic skeletal muscle damage leads to a need for greater regeneration, to examine whether hASM-AAV treatment reduces this need, we quantified the number of centrally nucleated myofibers, which was reduced by 2-fold (control-AAV, 38% ± 5.1%; hASM-AAV, 17.2% ± 3.7%; Figure 6, B and D, and Supplemental Figure 7, A and C), and concomitantly reversed the presence of small (regenerated) myofibers by approximately 45% in hASM-AAV–treated mice (Figure 6, B and E). Further, hASM-AAV–treated muscles showed a nearly 3-fold reduction in muscle fibrosis (Masson’s trichrome staining; Figure 6, F and G, and Supplemental Figure 7, E and F), and adipogenic loss of the myofibers (perilipin-1 staining; Figure 6, I and J, and Supplemental Figure 7, F and G). Finally, we examined whether these histopathological improvements by hASM-AAV treatment led to any improvement in muscle strength. We have previously found that dysferlin deficit causes greater force loss in the hindlimb muscles (54), and improved membrane repair addresses this deficit (36). Thus, we measured the forelimb and hindlimb muscle grip strength of the control-AAV– and hASM-AAV–treated mice. We found that compared with the control-AAV–treated cohort, while forelimb grip strength was not appreciably altered, the hindlimb grip strength was significantly improved in the hASM-AAV–treated cohort (Figure 6, H and K). In light of these findings, it is apparent that single-dose hASM-AAV treatment holds the potential to allow extended histopathological and functional muscle improvement for LGMD2B.

## Discussion

Restoration of the cellular deficits downstream of the lack of dysferlin protein can be a potential therapeutic approach for LGMD2B. To complement the ongoing gene therapy efforts aimed at restoring the expression of the large dysferlin protein in LGMD2B patient muscle, our work here provides an alternative approach. Using liver-targeted expression of a protein (ASM) nearly 4 times smaller than dysferlin, we address the downstream consequence of dysferlin deficit. This offered preclinical benefits comparable to skeletal muscle dysferlin restoration. We previously showed that dysferlin enables rapid and efficient lysosomal exocytosis required for timely secretion of ASM to help injured muscle cells repair frequent membrane injuries (10). Insufficient ASM release by injured cells is a deficit common to both LGMD2B and NPDA patients (10, 26). However, we find that unlike NPDA, dysferlin-deficient muscles do not lack ASM expression (Supplemental Figure 4). Increased extracellular hASM improves muscle health in the LGMD2B mouse model by improving plasma membrane repair through enhanced CLIC-mediated endocytosis (Figures 2 and 3). CLICs facilitate dysferlin endocytosis (44), localize with pore-forming toxins bound to the plasma membrane (30), and as demonstrated here, CLIC endocytosis is intricately linked with ASM-mediated plasma membrane repair (29).

Exogenous administration of hASM is safe for human use and shows therapeutic efficacy in treating symptoms caused by ASM deficit in NPD patients (39, 55). However, such studies have not assessed the capacity of hASM to improve membrane repair or evaluate its efficacy in treating LGMD2B — a disease caused not by the lack of ASM production, but by its reduced secretion. Our studies here have examined the reparative properties of hASM and identified the efficacious extracellular dose of hASM that can restore membrane repair capacity in dysferlin-deficient muscle cells (Figure 1). We found this dose to be lower than the dose that was used to enhance repair of ASM-deficient cells injured by pore-forming toxins (25). This hASM dose that is efficacious at improving plasma membrane repair is encouraging for its clinical utility in LGMD2B, as it is well below the established safe maximal hASM dose for use in humans and 100-fold lower than the dose that induces cell death (Supplemental Figure 5 and ref. 56). However, given the short circulating half-life of injected hASM protein (21–24 hours), therapy relying on direct hASM delivery will require frequent administration and dose escalation of the drug to maintain efficacy, thereby decreasing its utility in treating a chronic disease such as LGMD2B. To overcome this limitation, we tested a more clinically feasible approach of genetic delivery of hASM via hepatic expression of this protein by AAV-mediated delivery. With an excellent safety profile and high transduction efficiency of AAVs in a broad range of tissues, there are over 2,000 clinical trials to date that utilize these gene transfer vectors (57). Of these, liver-targeted AAV-based therapeutics offer greater efficacy of targeting by intravenous administration, allow multi-year transgene expression after single administration, and are efficient at treating plasma protein deficiencies (58–61). Despite the safety of this approach, a recent phase I/II clinical trial (NCT03199469) for X-linked myotubular myopathy (MTM) utilizing AAV8 dose escalation of MTM1 protein (AT132) observed detrimental hepatic outcomes in humans when high viral loads (1 × 10^14^ vg/kg up to 3 × 10^14^ vg/kg) were used. Our study shows therapeutic efficacy of hASM-AAV in mice at 1 × 10^13^ vg/kg (1 × 10^12^ vg/kg human equivalent dose). This significantly lower dose may enable safe use of the gene therapy approach we have outlined. Supporting this safety, we did not observe signs of liver damage (increased serum ALT level and liver histopathology) over the 12-week treatment with hASM-AAV.

Use of hASM-AAV in vitro showed that it allows production of secreted hASM by human liver cells (HepG2 cells) at levels that reached therapeutically efficacious concentrations and restores repair in dysferlin-deficient patient muscle cells (Figure 4). This efficacy is also reflected by the in vivo use of this vector in a preclinical mouse model of dysferlin deficiency. Use of this vector in the mouse model of NPDA has previously demonstrated increased and stable hASM production in a 12-week study (45). In our study, we confirmed this in vivo potential of hASM-AAV using the LGMD2B mouse model where there were detectable high levels of hepatic and serum hASM that were efficacious in restoring myofiber repair capacity 12 weeks after a single dose of this vector (Figure 5). These findings, while of interest for LGMD2B, is also of interest to NPDA patients since mouse models for NPDA also manifest poor sarcolemmal repair (26).

Increased muscle degeneration necessitates greater muscle regeneration, and we find that improved repair of dysferlin-deficient myofibers by hASM-AAV reduces the need for regeneration, causing a 2-fold decrease in the number of regenerated myofibers. It also decreased the proportion of small (newly regenerated) myofibers in the hASM-AAV–treated mice (Figure 6). These in vivo improvements by hASM-AAV are comparable to what is obtained by an AAV–mediated dysferlin gene therapy approach (32), and demonstrate that a secreted hASM–based gene therapy is as effective in rescuing the LGMD2B myofiber repair deficit as gene therapy that restored myofiber dysferlin expression. Additional consequences of persistent myofiber damage include chronic muscle inflammation (62). We find that hASM-AAV treatment of dysferlin-deficient mice also attenuated this, arguably through the improved in vivo repair ability of the dysferlin-deficient myofibers (Figure 6).

Continuous bouts of injury and poor membrane repair also promote fibroadipogenic replacement of muscle. We find that hASM-AAV caused reduced fibroadipogenic replacement of the dysferlinopathic muscle to an extent comparable to the reduction achieved using AAV–dysferlin gene therapy (ref. 32 and Figure 6). These findings indicated that the hASM-AAV treatment improves muscle repair and overall muscle quality and health, and should concomitantly preserve muscle function. In line with these improvements, we observed increased hindlimb grip strength following hASM-AAV treatment. Forelimb grip strength, which is known to be unaffected in the LGMD2B mouse model (63), was unaltered by the hASM-AAV treatment. Further, the observed improvement in hindlimb grip strength is in line with other preclinical approaches that offer therapeutic benefits for dysferlinopathy (64–66).

In summary, the results reported here demonstrate that hASM protein improves LGMD2B muscle cell sarcolemmal repair in a dose-dependent manner. They establish both purified hASM protein and AAV-mediated hepatic hASM gene transfer approaches as viable strategies for improving repair capacity of dysferlinopathic myofibers. Use of the gene transfer approach establishes its utility for longer-term in vivo benefits for reducing myofiber death and histopathology, as well as improving muscle function. Clinical trials are needed to examine the therapeutic utility of this approach to treat muscle pathology in LGMD2B and NPDA patients.

## Methods

### Animals.

B6.A-Dysf^prmd^/GeneJ (B6A/J) mice were purchased from The Jackson Laboratory and maintained in the animal house of the Children’s Research Institute (CRI). Animals were housed in a germ-free facility under a controlled 12-hour light/12-hour dark cycle with free access to food and water. Animals were genotyped before use in the experiments.

### Cell culture and treatments.

Immortalized control (healthy donor) and LGMD2B patient (with homozygous c.4882G mutation, leading to loss of any detectable dysferlin protein) myoblasts used were as described previously (10). Myoblasts were cultured in human myoblast culture media (PromoCell), supplemented with 10% FBS, on 0.4% gelatin–coated dishes and maintained at 37°C and 5% CO_2_. HepG2 cells and the C2C12 myoblast line (obtained from ATCC, HB-8065 and CRL-1772, respectively) were cultured in high-glucose DMEM supplemented with 10% FBS and 1% penicillin/streptomycin. For laser injury, cells were plated on fibronectin-coated glass coverslips. The cells were either injured as such or preincubated in cell imaging media (CIM; HBSS with 10 mM HEPES, 1 mM CaCl_2_, pH 7.4) for 20 minutes with varying concentrations of purified hASM (R&D Systems), or in culture supernatant of HepG2 cells transduced with hASM-AAV or control (eGFP-AAV) viral particles (for details of viral construct generation and treatment, see below). The cells were laser injured in CIM containing 1 μg/μL FM 1-43 dye (Life Technologies) and the same concentrations of hASM and cell supernatant as in the incubation period. Injury and subsequent imaging were performed at 37°C in the stage-top ZILCS incubator (Tokai Hit Co.). A 1- to 5-μm^2^ area of plasma membrane was irradiated for less than 10 ms with a pulsed laser (Ablate!, 3i Intelligent Imaging Innovations, Inc.) and cells were imaged at 2-second intervals with a 60×/1.45 NA oil objective on an Olympus IX81 microscope equipped with a 488 nm diode laser (Cobolt). FM dye intensity (*F*/*F_0_*, where *F_0_* is the original intensity) was quantified and repair was indicated by the block of FM entry leading to an increase in FM dye fluorescence, as described previously (67).

### Endocytosis assays.

For bulk endocytosis, plasma membranes of myoblasts (~70% confluent) were labeled with Alexa Fluor 488–conjugated WGA (3 μg/mL) for 2 minutes at 37°C. After washing the excess WGA with CIM, cells were left untreated or treated with hASM (6 U/L in CIM), and imaged using a 40×/1.4 NA or 60×/1.45 NA oil objective on an Olympus IX81 microscope, simultaneously in widefield and confocal modes. WGA endocytosis was allowed and at different time points bromophenol blue (BPB) was injected into the imaging chamber (final concentration of 4 mM) to quench WGA at the cell surface. To assess extent of membrane endocytosis, following background correction, the average postquench fluorescence of each cell was divided by its initial prequench fluorescence, and normalized to the fraction of internalized membrane assessed after immediate quenching (0-minute endocytosis).

For caveolar endocytosis, cells transfected with mRFP-tagged caveolin-1 were imaged as described previously (ref. 68 and Supplemental Figure 1A). Cells were imaged in CIM with a 60×/1.45 NA oil objective as described above, using an Olympus IX81 microscope equipped using a 560 nm confocal diode laser (Cobolt), at the membrane-coverslip interface. Cells were imaged at 1 Hz as indicated. To quantify caveolin mobility, 50 individual caveolin puncta/vesicles were marked in each cell at the start of imaging. Each vesicle was subsequently tracked manually. A vesicle was deemed mobile if it either migrated laterally for a distance of greater than 1.5 μm or moved axially such that it was absent from the imaging plane for 10 seconds or longer, or both. The fraction of vesicles (out of 50 for each cell) was quantified for the 2-minute time point.

For CLIC/GEEC (CLIC/GPI-anchored protein–enriched compartments) endocytosis assays, cells were transfected with GPI-GFP (69). Transfected cells were imaged as above at a *z* plane through the middle of the cell body at 1 frame per minute for 20 minutes. As needed, hASM was added to the chamber after the second image. GPI-GFP membrane fluorescence was monitored by marking plasma membranes and correcting for photobleaching (Supplemental Figure 1C). Endocytic rates were obtained by curve fitting the membrane fluorescence kinetics trace spanning the time point of interest, and using this to calculate the rate of loss of membrane fluorescence at that specific time point. Images were quantified using SlideBook 6.0 (Intelligent Imaging Innovations, Inc.).

### Membrane shedding assay.

C2C12 cells (at ~50% confluence) were labeled with FITC-PEG-cholesterol (5 μM; PEG-2000, Nanocs Inc., PG2-CSFC-2k) for 30 minutes at 37°C in CIM (Supplemental Figure 1B). After washing the excess label, cells were immediately imaged in CIM by simultaneous confocal and widefield microscopy, with a 60×/1.45 NA oil objective on an Olympus IX81 microscope equipped with a 488 nm diode laser. Cells were imaged at 0.2 Hz for 2 minutes. As needed, hASM was added approximately 20 to 30 seconds prior to onset of time-lapse acquisition. The images were acquired at a *z* plane positioned at the cell-coverslip interface to monitor vesicle shedding on the surrounding coverslip area. Vesicles were quantified using MetaMorph 7.0 (Molecular Devices) in a 5,000-μm^2^ area on the coverslip surface adjacent to the cell (sum of vesicles shed over the 2-minute period) and normalized to vesicles present at the onset of acquisition. To assess the loss of cellular fluorescence, widefield images were corrected for photobleaching, followed by analysis of the loss of fluorescence in 2-minute period, using SlideBook 6.0 software.

### Western blotting and immunostaining.

HepG2 cell lysates were resolved in 4% to 12% gradient polyacrylamide gels, transferred to nitrocellulose membranes, and probed with antibodies against ASM (rabbit polyclonal, Abcam, ab83354) and β-actin (mouse monoclonal, Santa Cruz Biotechnology, sc47778). Primary antibodies were followed by the appropriate HRP-conjugated secondary antibodies (Sigma-Aldrich) and chemiluminescent Western blotting substrate (GE Healthcare) and processed on a ChemiDoc MP system (Bio-Rad Laboratories).

### AAV vector generation and delivery.

For AAV8/DC190-hASM vector production, construction of the previral plasmid carrying hASM cDNA has been described previously (45). Briefly, expression of the hASM cDNA (NM_000543) is driven from the liver-restricted promoter/enhancer DC190 (human serum albumin promoter/α1-microglobulin enhancers). The expression cassette also contains a hybrid intron. The polyadenylation signal is followed by a fragment of the human α1-antitrypsin intron, bringing the size of the recombinant viral DNA to approximately 4.5 kb for optimal packaging. Plasmid DNA was purified using a Qiagen EndoFree Plasmid purification kit. The AAV2-based previral plasmid was packaged into AAV serotype 8 capsids. Recombinant AAV virus was produced by triple plasmid transfection followed by cesium chloride density gradient purification by the University of Massachusetts Medical School Vector Core Gene Therapy Center. Genome copy titers of the AAV vectors were determined using a real-time TaqMan PCR assay (ABI Prism 7700; Applied Biosystems) with primers that were specific for the bovine growth hormone (bGH) polyadenylation signal sequence. AAV9.CMV.PI.eGFP.WPRE.bGH (lot CS0273) was used as the control AAV vector (Vector Core at the Perelman School of Medicine, University of Pennsylvania). Viral particles were stored as suspension in sterile PBS with 5% glycerol at –80°C. The viral particle suspension was thawed, diluted, and delivered via intravenous administration at a viral dose of 3.4 × 10^11^ particles per mouse or 1.1 × 10^13^ vg/kg. Mice used for this study were derived from 2 separate litters of BLA/J mice consisting of a mixture of male and female mice that were born on the same day. Each pup was identified by ear-tag ID, and a random draw from each litter was based on coded ID numbers to ensure that (a) mice from both litters were allocated to each treatment group and (b) both male and female mice were represented in each treatment group. In the hASM-AAV group, 5 mice were injected with hASM-AAV. The control group having the same number of mice was injected with control-AAV. After the injection, experimental mice were kept in the home cage for 3 months until experimentation.

### ASM measurement.

Livers and quadriceps muscle were snap frozen in liquid nitrogen–cooled isopentane (and stored at –80°C), while serum collected via retro-orbital bleeding at baseline, 1, 4, and 12 weeks after injection was stored at –80°C. For assays, tissue samples were ground and homogenized with a microtube homogenizer in RIPA buffer (Sigma-Aldrich) plus protease inhibitor cocktail (Thermo Fisher Scientific) on ice. Lysates were assessed for total protein concentration using a BCA protein assay and plate reader. Equal amounts of total lysate protein (4.1 μg for liver, 25 μg for quadriceps muscle) and serum volume (5 μL), were used across all samples for determination of hASM activity using the Amplex Red Sphingomyelinase assay kit (Invitrogen). All samples were run in triplicate. Activity was thus expressed as units of activity (hydrolyzing activity, U) per gram of liver and muscle tissue (for liver/muscle ASM activity), and U/L serum. Activity was averaged across the 5 samples per treatment condition and are expressed as mean ± SEM.

### Serum ALT concentration.

Serum (5 μL) from each of the above-listed time points after AAV injection was assayed for ALT concentration, a marker of liver damage/disease, using a colorimetric assay (Cayman Chemical) according to the manufacturer’s instructions. All samples were run in triplicate, with the ALT concentration averaged across all samples per treatment condition, per time point, and are expressed as mean ± SEM.

### hASM-AAV–mediated in vitro hASM production and quantification.

HepG2 cells in a 96-well dish at a density of approximately 1 × 10^5^ cells/well were infected in antibiotic-free DMEM with 4.5 × 10^6^ particles of Ad5 (multiplicity of infection [MOI] of 45 particles/cell) for 2 hours. Cells were infected with AAV2/8 DC190-hASM or control vector at 1 × 10^10^ genome copies/mL (MOI of 1 × 10^4^) in a volume of 100 μL for 1 hour, at which point 100 μL of complete DMEM was added. On day 5, the cell culture media were collected and used immediately for subsequent experiments or stored at –80°C. Cells were pelleted by scraping in ice-cooled PBS and lysed with RIPA buffer (Sigma-Aldrich) containing protease inhibitor cocktail (Thermo Fisher Scientific). The culture supernatant and cell lysates were used for fluorimetric assay of hASM activity using the Amplex Red Sphingomyelinase assay kit (Invitrogen) and for Western blots. ASM kinetics was analyzed over the course of 20 minutes using an EnSpire Multimode Plate Reader (PerkinElmer) with fluorescence emission detection at 585 nm. hASM activity is thus expressed in units of activity per liter of supernatant, or gram of cell lysate. All samples were assessed in triplicate and a standard curve was generated (Supplemental Figure 2).

### hASM cellular toxicity in vitro.

Healthy donor myoblasts were cultured in 0.4% gelatin–coated 6 cm culture dishes, and were grown to 60% confluence in human myoblast culture media (PromoCell) supplemented with 10% FBS and maintained at 37°C and 5% CO_2_. Upon reaching 60% confluence, growth media were supplemented with titrated concentrations of hASM protein (control [PBS], 8, 80, and 800 U/L) for 24 hours. Subsequently, cells were collected and assessed for cell viability/death via trypan blue assay, with cell death expressed as a percentage of total cells. Cell death experiments were conducted with 3 biological replicates per hASM dosage.

### Histology and immunohistochemistry.

Transverse cryosections (8 μm thick) of the quadriceps muscle and liver were prepared using a CM3050S cryostat (Leica Biosystems) and stored at –20°C for later staining (*n =* 5 per group). After thawing, muscle sections were processed for H&E, laminin (1:100; anti–laminin-2 α-chain, rat monoclonal, Sigma-Aldrich, clone 4H8-2), anti-IgM (1:100, Invitrogen, A-31552), anti-perilipin (1:250; Sigma-Aldrich, P1873), Masson’s trichrome (Trichrome Stain Kit, Abcam), while liver sections were processed for H&E only. Images were captured with a VS120 slide-scanning microscope (Olympus America) at ×40 magnification, and quantified using CellSens software (Olympus America). For immunostaining, muscle sections were blocked in 5% BSA for 1 hour (laminin staining) or 1% BSA, 10% goat serum, and 0.1% Tween (for perilipin). Alexa Fluor 488– or 594–conjugated (1:500) secondary antibodies were used and costained with WGA and DAPI.

To quantify muscle inflammation, clusters of extramyofibrillar nuclei consisting of more than 9 nuclei were noted as inflammatory foci, and quantified from either the entire quadriceps cross section or specifically within the rectus femoris muscle and vastus muscles as noted in H&E-stained sections, and are expressed per mm^2^ cross-sectional area. These sections were also used to quantify centrally nucleated fibers, which are expressed as a percentage of total myofibers counted per muscle section (whole quadriceps cross section, rectus femoris, and vastus regions). Centrally nucleated myofiber counts were independently verified using laminin and DAPI costained sections and the CellProfiler Muscle Analyzer pipeline, as described previously (70). The same pipeline was used to assess myofiber cross-sectional area across 3 mice per group, for a total of 3,000 fibers per group, and measured in μm^2^. Muscle fibrosis/collagen accumulation was quantified using Masson’s trichrome staining. Five representative images per quadriceps cross section were obtained from the whole muscle image, and assessed for percentage of total muscle area taken up by stained collagen tissue (stained blue), using ImageJ (NIH) as described previously (71). Selected images were split into red, blue, and green channels, with subsequent thresholding for the blue channel image to quantify collagen-stained fibrotic tissue.

For quantification of in vivo–injured myofibers, the total number of WGA-labeled fibers from the entire quadriceps cross section was scored for fibers that were positive for IgM. These were then presented as the number of IgM-positive fibers per mm^2^ cross-sectional area of the muscle. To quantify adipogenic deposits, anti-perilipin–stained quadriceps muscle sections were assessed using MetaMorph software and are presented as percentage of perilipin-positive area (for whole quadriceps, rectus femoris muscle, and vastus). For the liver histopathology scoring, H&E-stained sections were scored for features such as hepatocyte necrosis, apoptosis, karyolysis, degeneration, loss (focal or diffuse), vacuolation, hypertrophy, fibrosis, and inflammation on a scale of 1 to 5 (higher scores indicating worse pathology) by a trained, blinded veterinarian. Each liver sample score was averaged from 5 representative fields per liver section.

### Grip-strength measurement.

Forelimb and hindlimb grip-strength measurement was assessed using a grip-strength meter (Columbus Instruments), as previously described (72). The animals were acclimatized for 3 days before data collection. The forelimb and hindlimb grip-strength data were then collected over 5 consecutive days, and are represented as averaged grip strength/kg body weight over 5 days, as previously described (36).

### Ex vivo myofiber injury.

For contraction-induced sarcolemmal injury, EDL muscles were extracted from WT BL6 or from B6A/J mice treated with hASM-AAV or control-AAV, and placed in Ringer’s solution (137 mM NaCl, 24 mM NaHCO_3_, 11 mM glucose, 5 mM KCl, 2 mM CaCl_2_, 1 mM MgSO_4_, 1 mM NaH_2_PO_4_, and 0.025 mM tubocurarine chloride) bubbled with 95% O_2_ and 5% CO_2_ to maintain pH at 7.4. The distal tendon was securely connected to a fixed-bottom plate, and the proximal tendon was attached to the arm of a servomotor (800 A in vitro muscle apparatus, Aurora Scientific) with 6-0 silk sutures. The vertically aligned EDL muscle was flanked by 2 stainless steel plate electrodes. Using single 0.2-mm square simulation pulses, the muscle was adjusted to the optimal muscle length for force generation. At optimal length, with isometric tetanic contractions 300 ms in duration at frequencies up to 250 Hz separated by 2-minute rest intervals, the maximal force was determined. Contraction-induced sarcolemma damage was induced by 9 sequential lengthening contractions (LCs) with 10% strain at a velocity of 2 fiber lengths per second. Each contraction was separated by a 1-minute rest interval. LC-induced force loss was expressed as percentage of first contraction. At the end of LC protocol, muscles were trimmed of tendons, blotted, weighed, and incubated in a 0.2% PO solution at room temperature for 30 minutes. After washing the excess dye, the tissue was snap frozen in liquid nitrogen–cooled isopentane prior to being sectioned and imaged for PO-labeled fibers, with unlabeled tissue being used to determine background fluorescence. The number of PO-positive myofibers was expressed as a percentage relative to the total myofibers in the muscle cross section and fibers at the edge of the sections were excluded from analysis. For focal laser injury assay, intact biceps muscles were mounted in prewarmed Tyrode’s buffer (119 mM NaCl, 5 mM KCl, 25 mM HEPES, 2 mM CaCl_2_, 2 mM MgCl_2_, 6 g/L glucose, pH 7.4), with FM 1-43 dye (1–2 mg/mL) and imaged using the 40×/1.4 NA the Olympus IX81 microscope as described for cell laser injuries above. Repair kinetics and successful myofiber repair were determined as described previously for cell injury assays (16).

### Study rigor.

A priori sample size determination for the in vivo portion of this study was derived from 2 prior studies conducted in our lab assessing the proreparative effect of membrane lipid–stabilizing drugs (bacterial sphingomyelinase and vamorolone; refs. 10, 36). For laser ablation injury assessment of repair capacity, we carried out a power analysis from our vamorolone trials, and found an effect size of 0.725 with this membrane lipid–modifying drug. With a 2-tailed α set at 0.05 and power at 80%, this dictates that 5 mice per treatment group are required to achieve statistical significance. Similarly, bacterial sphingomyelinase improved myofiber membrane repair capacity in our prior studies with an effect size of 0.6, requiring the use of 6 mice per group to assess significant effect on repair capacity assuming 2-tailed α of 0.05 and power at 80%. Thus, upon compiling these prior data from studies examining the effects of compounds or drugs that modify cell membrane lipids in LGMD2B (BLA/J mice), as hASM does, we ascertained that we would require 5 mice per group for our primary endpoint measure (membrane repair capacity) and 4 to 7 mice per treatment group to find statistically significant differences for additional endpoints tested (see supplemental methods for sample size determination for other endpoints).

All in vivo measures (laser injury assays, all muscle and liver histology measures, ASM and ALT activity, eccentric force assay) were obtained by a blinded member of the research team. Blinding was accomplished through the use of a deidentifying code sheet that contained mouse ear-tag number and treatment group. The repair assays shown in Figure 1 and Figure 4, D and E, were coded to blind the rater/data analyzer to condition. Assays involving added recombinant hASM were conducted by an unblinded team member, but the rater was blind to sample identity for in vitro ASM activity assays (Figure 4, B and C).

### Statistics.

For cell injury and biceps myofiber repair kinetics (FM dye intensity kinetics), eccentric force decrement traces, and CLIC/GEEC endocytosis kinetics, all generated curves were compared via mixed-model ANOVA with analyses for interaction effects between the main effects of treatment condition and time or trial. In the event of significant interaction, group differences in FM dye fluorescence intensity/membrane fluorescence/eccentric force were assessed per time point via Holm-Sidak test, and Huynh-Feldt correction due to violation of sphericity. One-way ANOVA was used to determine differences in the number of cells and/or myofibers that failed to repair following injury, and in general membrane endocytosis measures. Repeated-measures ANOVA was used to assess for differences in body weight changes over the 12-week treatment period, and in CLIC/GEEC endocytosis rates, between conditions. Comparisons between control-AAV– and hASM-AAV–treated mice in hepatic ASM production, serum ASM activity, serum ALT concentration, proportion of fibers that repair with injury, histology measures (IgM^+^ proportion, Masson’s trichrome staining for fibrotic area, inflammatory foci, central nucleation, perilipin^+^ proportion, PO^+^ proportion, and myofiber area) and limb force measurements were calculated using independent samples *t* test. Similarly, independent samples *t* tests were used to calculate differences in ASM activity of transfected HepG2 cells (both cell supernatants and lysates), in the C2C12 caveolin endocytosis mobile fraction, and membrane shedding measure (untreated vs. hASM-treated). For all statistical analysis, α level was set at *P* less than 0.05.

### Study approval.

All animal procedures were reviewed and approved by the Institutional Animal Care and Use Committee of Children’s National Hospital (CNH) in Washington, DC.

## Author contributions

JKJ, GC, SCS, and DCB conceived and designed the study and RZ generated the AAVs used. DCB, SCS, GC, JHVDM, KN, and JKJ acquired, analyzed, or interpreted data. JKJ obtained funding and JKJ and DCB drafted the manuscript, which was edited by all the authors.

## Supplementary Material

Supplemental data

Supplemental video 1

Supplemental video 2

Supplemental video 3

Supplemental video 4

## Version 1 01/04/2022

Electronic publication
