# Supplementary material for: Secreted acid sphingomyelinase as a potential gene therapy for limb girdle muscular dystrophy 2B
Source: J Clin Invest. 2022 Jan 4;132(1):e141295. doi: 10.1172/JCI141295 (PMC8718136; doi:10.1172/JCI141295)
Supplement: Supplemental data [file jci-132-141295-s051.pdf]

Supplemental Figures

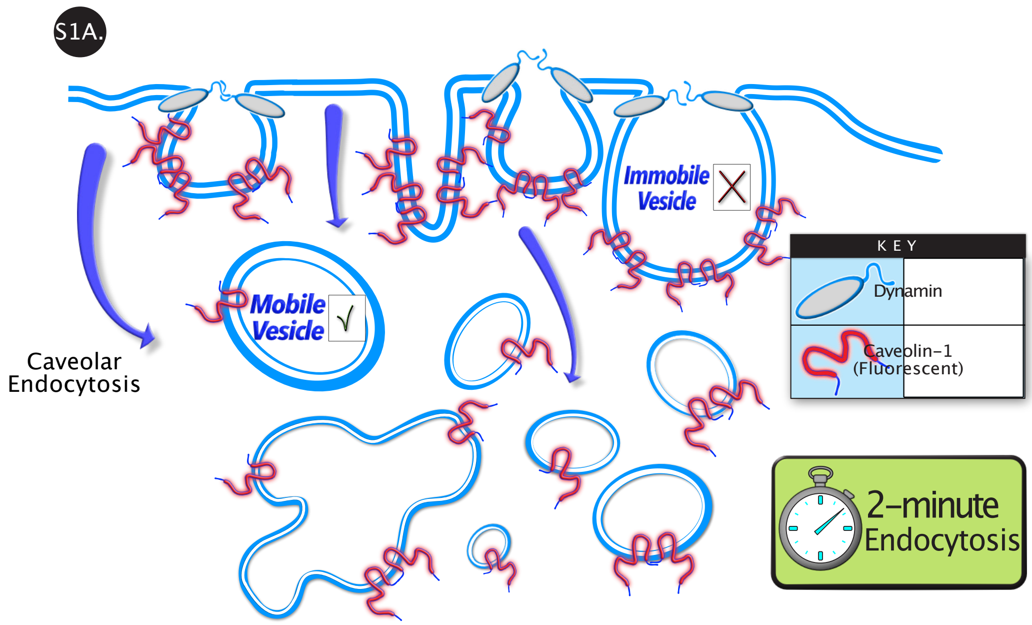

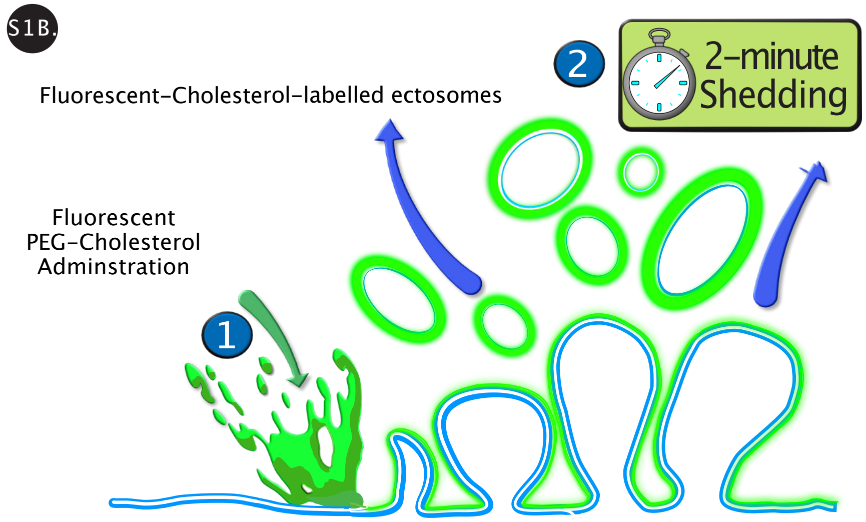

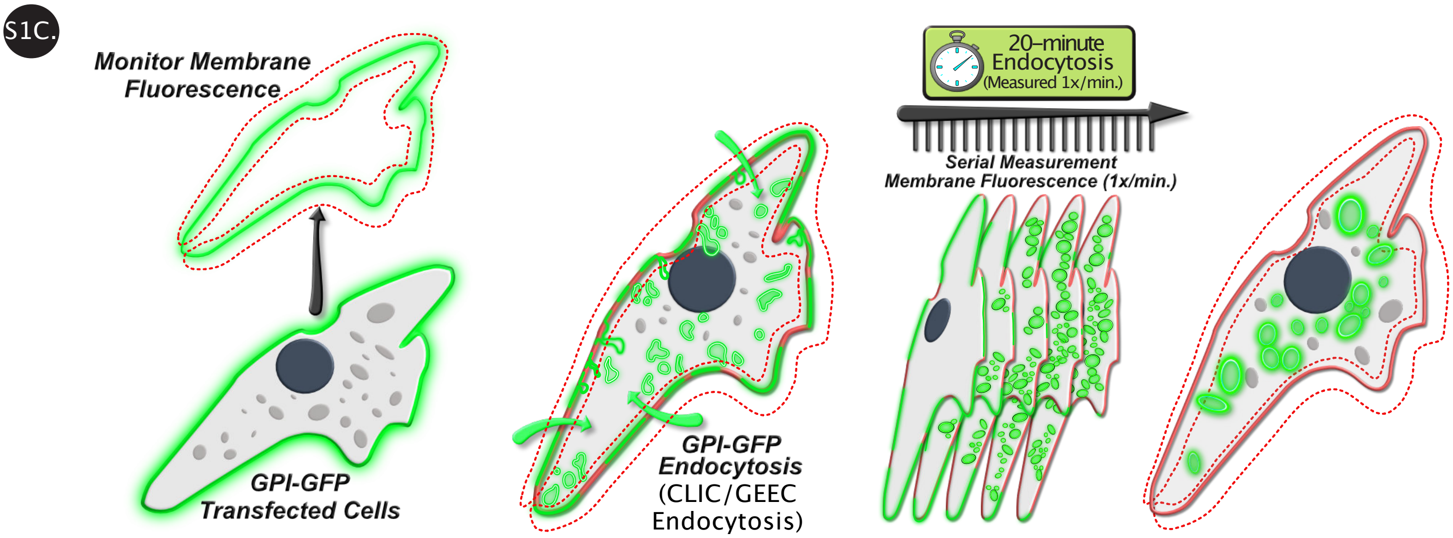

Figure S1. Schematic showing membrane trafficking assays

(A) Caveolar Endocytosis Activity. Caveolin-1+ puncta/vesicles from transfected myoblasts were tracked (via confocal-microscopy) over a 2-minute period for internalization/mobility. 50 individual caveolin puncta/vesicles were highlighted from each cell. Each vesicle was subsequently tracked manually over time to determine its mobility status. A vesicle was deemed mobile if it migrated laterally within the membrane (distance exceeding 1.5 um), was endocytosed (vesicle internalized and absent from the imaging plane for $\geq$ 10 seconds), or if a component vesicle of caveolin clusters was observed to exceed these movement parameters. Upon meeting these assigned movement-thresholds, a vesicle was marked as mobile, and the timeframe at which it exhibited mobility was noted. Percentage of mobile vesicles was then quantified for the 2-minute period, for each condition – termed mobile fraction (mobile-fraction = percent mobile vesicles relative to total, Untreated vs. hASM-treated).

(B) Membrane shedding assay. FITC-PEG-Cholesterol was inserted into the myoblast cell membrane. Membrane fluorescence and release of fluorescent-cholesterol-containing vesicles was tracked over 2-minutes. The confocal z-plane was positioned to monitor FITC-Cholesterol-enriched vesicle shedding/deposition onto the surrounding coverslip area, while widefield images were obtained to monitor change in cell fluorescence over the 2-minute period. For vesicle quantification, a 4,000-5,000 um^2 area of the surrounding coverslip surface was monitored for total vesicle secretion (sum of vesicles shed over the 2-minute period) and normalized to baseline/pre-existing vesicles present at the onset of image acquisition

(C) CLIC/GEEC Endocytosis. Fluorescent-GFP-tagged glycosylphosphatidylinositol anchors (GPI-GFP) were transfected into C2C12 myoblasts or Patient primary myoblasts (healthy, and LGMD2B), inducing membrane fluorescence. Membrane fluorescence was tracked over a 20-minute period via confocal microscopy (60X/1.45NA oil objective) focused on 1 z-plane slice. Images were acquired 1x/min. to track loss of membrane fluorescence, as a measure of CLIC/GEEC endocytosis of GPI-GFP. Rate of internalization was obtained from kinetics traces of membrane fluorescence loss, and compared between untreated and hASM-treated cells.

FIG. S2 – hASM activity assessment

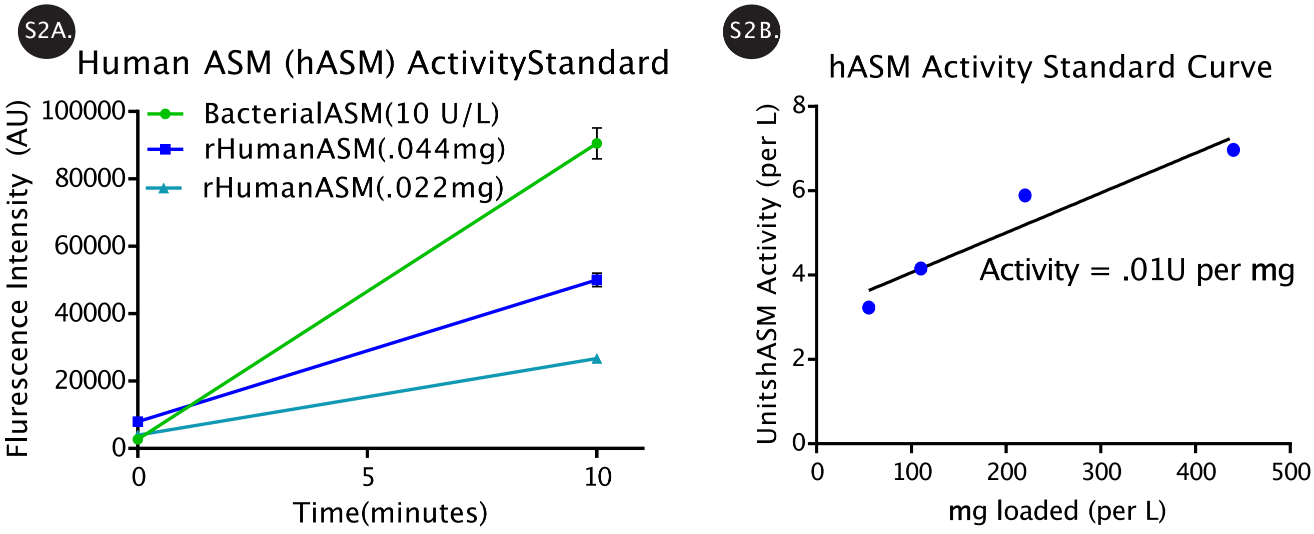

Figure S2. Recombinant hASM activity transposition
Recombinant human ASM (hASM) (R&D Systems, Minneapolis, MN, USA) was analyzed for its ASM activity using the Amplex Red Sphingomyelinase assay kit. **A.** Titrated amounts of total hASM were diluted in 1x reaction buffer to concentrations of 0.44 mg/L, 0.22 mg/L, .011 mg/L in wells of a 96-well plate. Dilutions were subsequently mixed with Amplex Red Reagent Cocktail (containing 2U/mL horseradish peroxidase + 0.2U/mL choline oxidase + 8 U/mL alkaline phosphatase + 0.5 mM sphingomyelin) as described in the kit-provided protocol. Positive controls included 10 uM H_2O_2 in 1x reaction buffer, and 10 U/mL activity bacterial sphingomyelinase stock diluted in 1x reaction buffer. All samples were analyzed for ASM kinetics over the course of 10 minutes (emission detection of 585 nm wavelength). All samples were assessed in triplicate. **B.** ASM activity of hASM was transposed from fluorescence emission values to units of activity using the known activity and fluorescence emission of the bacterial sphingomyelinase positive control (10 U/L) and the generated standard curve shown here. hASM protein has a units-of-ASM-activity conversion of .01 units per mg protein.

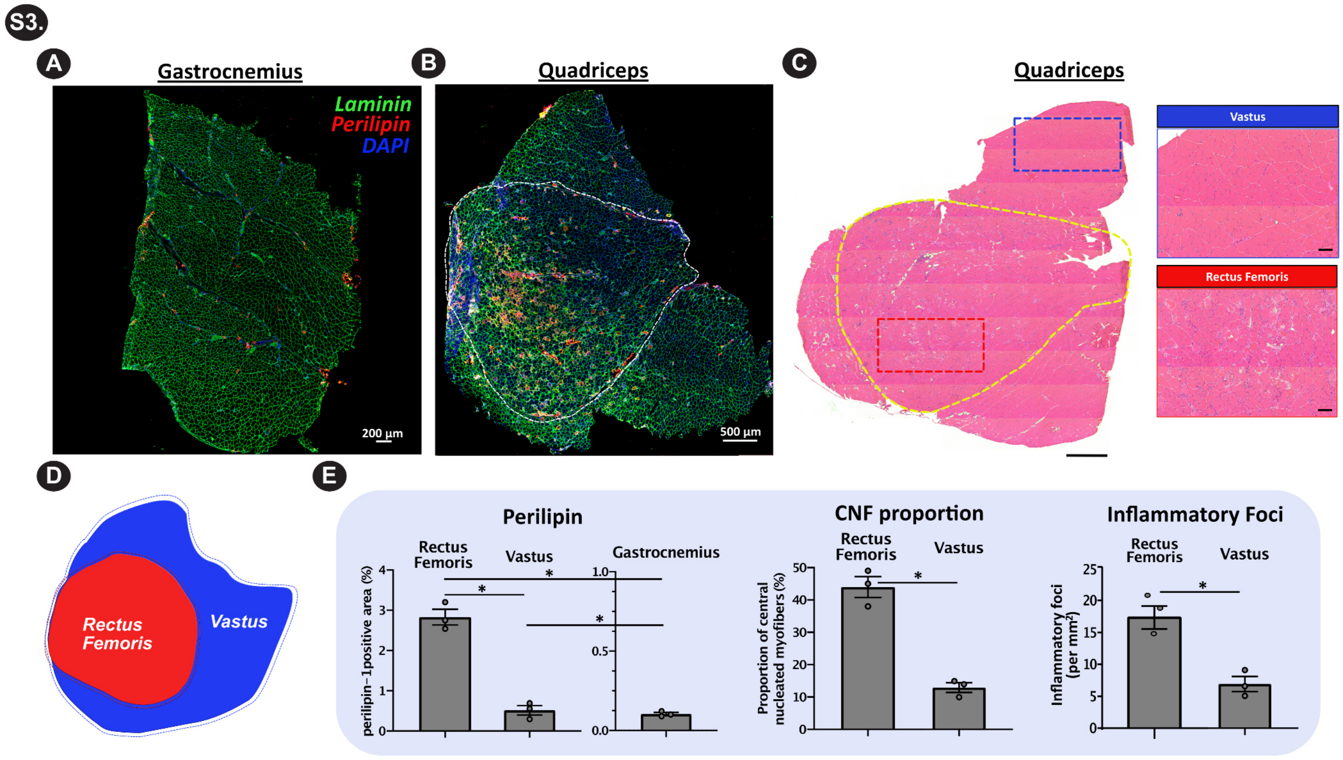

Figure S3. Muscle-specific histopathology 6-months-old mouse model of LGMD2B. Histological assessment of two of the lower limb muscle gastrocnemius and quadriceps was carried out in B6A/J mice at 6-months of age. **A.** Representative images of whole-gastrocnemius and **B.** Whole quadriceps cross-section immunostained for Laminin (green), Perilipin (red), and nuclei (blue). Rectus femoris muscle of the quadriceps is marked by white dotted line. **C.** Schematic depiction of quadriceps muscle anatomy - rectus femoris is centrally located (red), flanked by vastus lateralis, vastus medialis, and vastus intermedius – collectively termed vastus (blue) **D.** Hematoxylin-Eosin staining of whole quadriceps cross-section (rectus femoris marked by yellow dotted line). Zoomed in images represent a portion of rectus femoris (red dotted box; note the profuse inflammatory cell infiltrate, degeneration, and central nucleated myofibers) and of the vastus portion (blue dotted box; lacks above histopathology). **E.** Plots showing the quantification of percentage of (Left) perilipin positive cross-sectional area, (Center) total centrally nucleated fibers (CNF) (Right) number of inflammatory foci per mm^2 cross-sectional area. All * $p<.05$ vs. rectus femoris (via independent samples t-test). H&E scale bar (whole quadriceps = 500 m, inlay images 100 m).

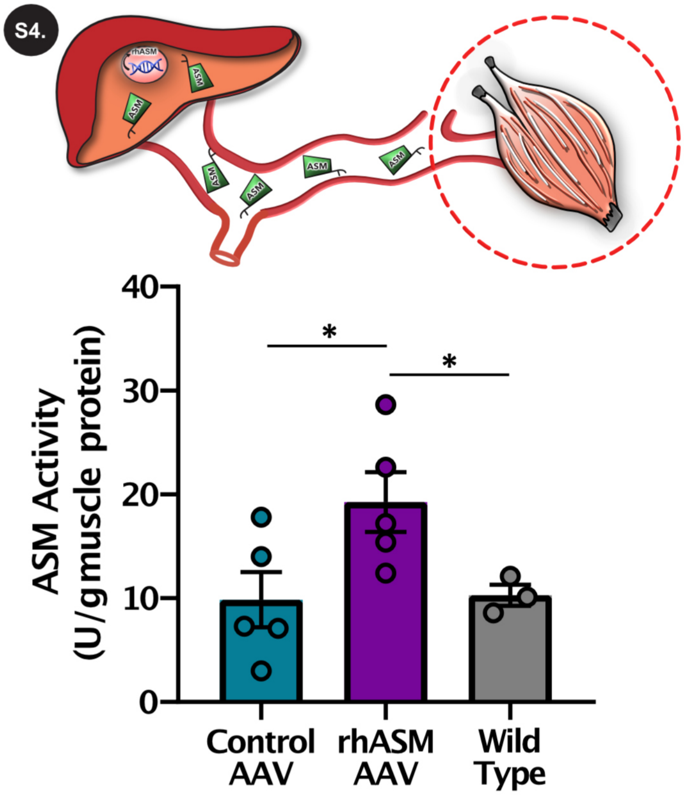

Figure S4. Recombinant hASM activity in skeletal muscle
Recombinant human ASM (hASM) (R&D Systems, Minneapolis, MN, USA) was analyzed for its ASM activity using the Amplex Red Sphingomyelinase assay kit. 5 ug of muscle protein (quadriceps) was utilized per mouse, per condition to hASM activity assessment (each sample analyzed in triplicate - Control-AAV, hASM-AAV, n = 5 mice per AAV group, and 3 per wild-type). Data is presented as mean hASM activity ± SEM. *p = 0.011 (vs. Control-AAV) by independent samples t-test.

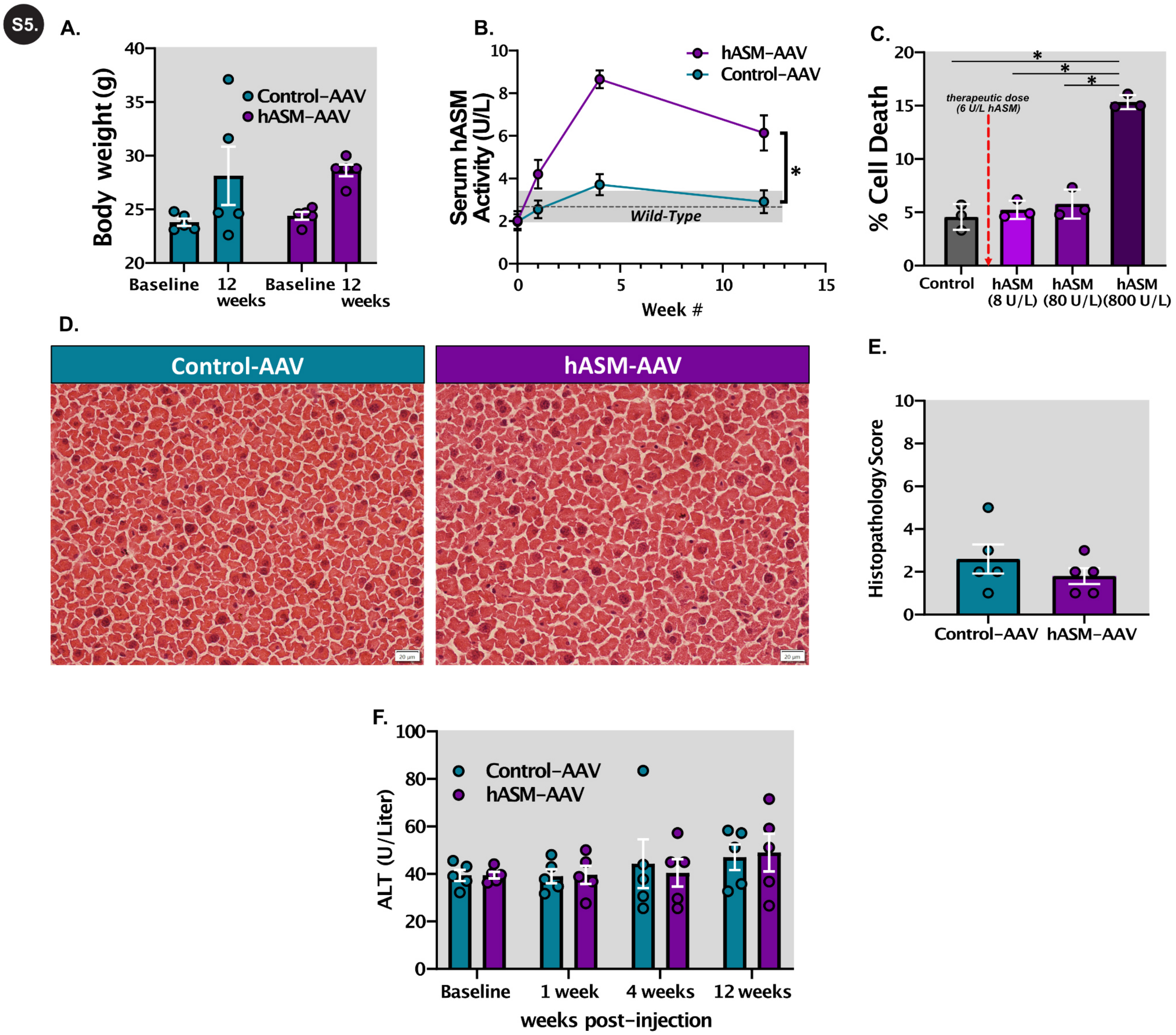

Figure S5. Effects of liver-targeted AAV on health, cell viability, and serum markers
A. Plot showing paired changes in bodyweight of AAV-treated mice from prior-to and 12-weeks after single AAV injection. **B.** Plot showing hASM activity in the serum of hASM-AAV and control-AAV injected mice at baseline (week 0), and at 1-, 4-, and 12-weeks post injection (expressed in U/L). Two 22-week-old wild-type BL6 mice were assessed for serum ASM activity as a comparison to normal levels (mean = dotted line, gray shading = min/max). **C.** Plot showing hASM cellular toxicity and concentration relationships. Human myoblasts were cultured in growth media supplemented with titrated concentrations of hASM protein (Control/PBS, 8, 80, 800 U/L) for 24 hours (6 U/L was the minimal therapeutic dose – denoted by the red line). Cells were collected and assessed for cell viability/death via trypan blue assay. Data is presented as mean proportion of cells that died (%) of total cell count. **D.** H&E histology images of representative liver sections from control- and hASM-AAV treated mice 12-weeks post single-injection. **E.** Plot of histology scores for mice from each treatment group with suitable/quality histology images that afforded histopathological grading (scale: 1-10, with higher scores indicating worse pathology. 3 = focal degeneration/necrosis, 4 = focal/extensive inflammation/degeneration/or necrosis, 5 = massive necrosis/hepatocyte loss). Not shown – PBS injected BLA/J mouse score of 3: focal degeneration and necrosis. **F.** Plot showing serum ALT levels across the 12-week study period, to assess the effects of the liver-targeted AAV on liver health and/or liver injury (normal range 5-month-old BL6 mice: ~22-40 U/L) (1). Histology images acquired at 40x magnification, scale bar = 20 um. 5 µL of serum was utilized per mouse, per

condition, per timepoint for both serum hASM activity and serum ALT assessment (each sample analyzed in triplicate - Control-AAV, hASM-AAV, n = 5 mice per AAV group, and 3 per wild-type in serum analysis). Data is presented as mean ± SEM. B. *p = 0.011 (vs. Control-AAV) by mixed model ANOVA with analyses for interaction effects between treatment condition and time, with post-hoc comparison at 12-week timepoint. Change in bodyweight between treatment groups (A) was assessed via repeated-measures ANOVA. hASM cellular toxicity (C) *p <.001 (vs. 800 U/L hASM) by independent 1-way ANOVA. Each datapoint represents independent experimental repeat (biological replicate).

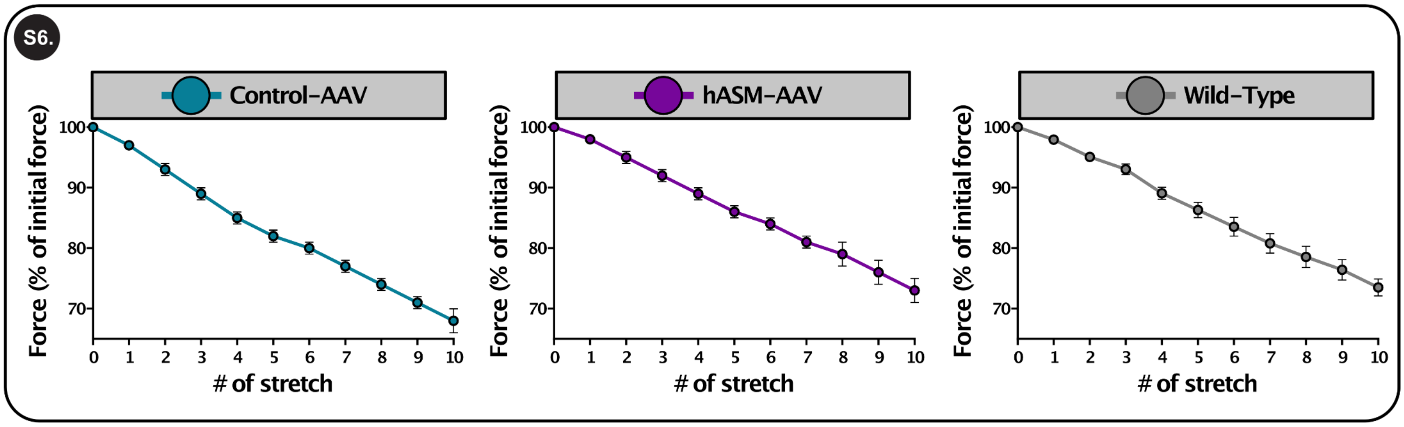

Figure S6. Effects of liver-targeted AAV on preservation of strength upon mechanical injury. 12 weeks after a single I.V. dose of liver-specific hASM-AAV or control-AAV (3.4×10^{13} vg/kg), extensor digitorum longus (EDL) muscles were isolated for functional measurements. Isolated EDL muscles from control-AAV and hASM-AAV treated mice were injured by stimulation of repeated 10% eccentric contractions to induce sarcolemmal damage, that if not adequately or efficiently repaired, may induce rapid impairment in muscle function. Plots demonstrate the change in muscle contractile force with each of 10 repeated eccentric contractions across the conditions (data is the same as presented in Fig. 5L, with traces separated to visualize wild-type force levels in comparison with control- or hASM-AAV treated muscle. *Note the restoration of eccentric force to hASM-AAV treated muscle, similar to wild-type (n=4 per group). Data is presented as mean ± SEM.

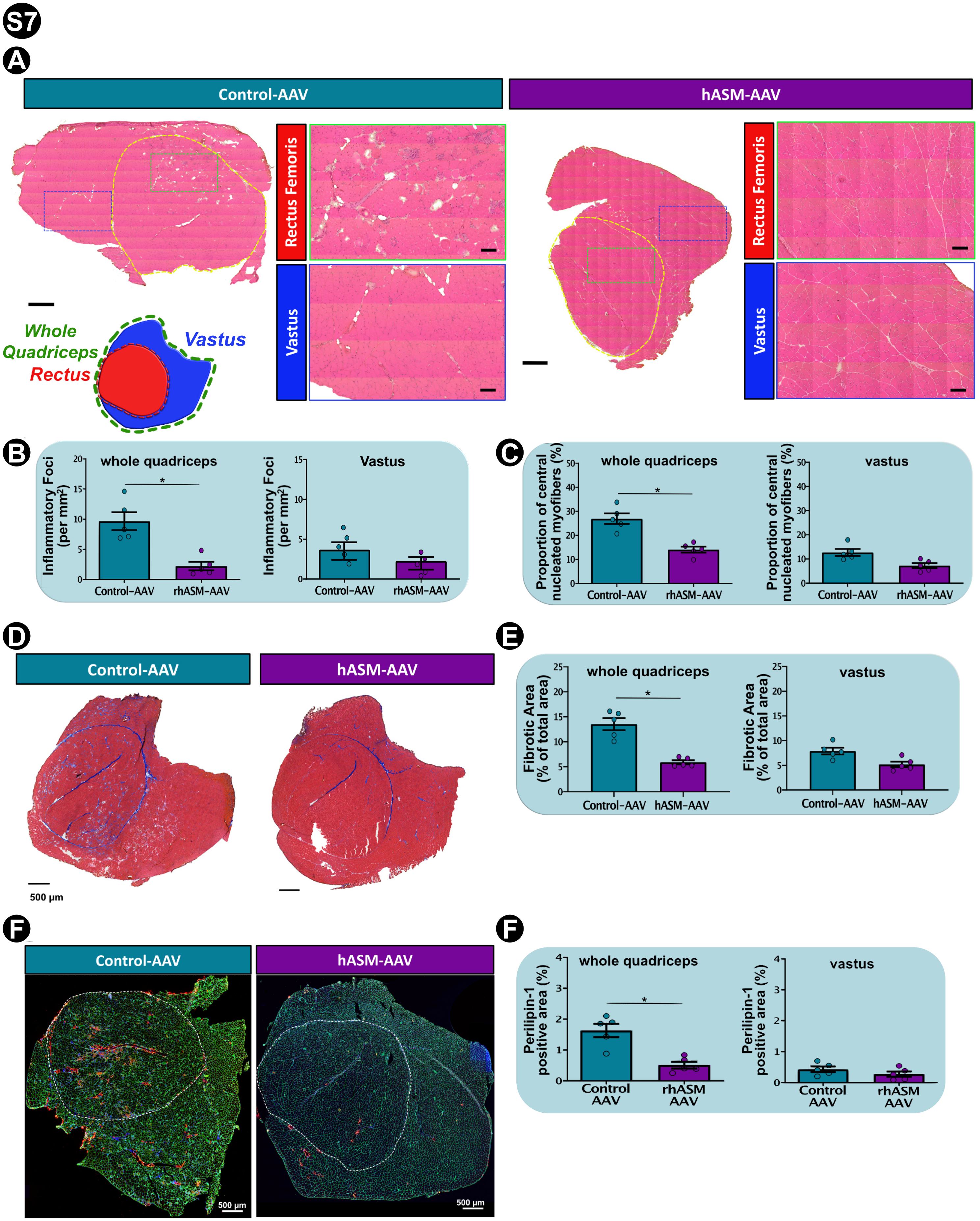

Figure S7. Effect of ASM-AAV therapy on quadriceps histopathology in B6A/J mice. (**A**) Images showing histopathology of H&E-stained quadriceps muscle of 6-month-old B6A/J mouse muscle treated for 12-weeks with hASM-AAV or control-AAV treatment. Rectus femoris muscle is outlined in yellow. and inlays show – green (rectus femoris muscle), blue (vastus muscle).

(**B**) Plot showing quantification of inflammatory foci (cluster of 9+ mononuclear cells) across the whole quadriceps (left plot) and vastus region (right plot). (**C**) Plot showing quantification of percentage of myofibers that are centrally nucleated across the entire quadriceps (left plot) and vastus region (right plot). (**D**) Images, (**E**) Quantification of Masson trichrome staining in entire quadriceps cross-section (left plot), or just the vastus muscle group (right plot). (**F**) Images showing whole-quadriceps cross-sections of 6-months-old B6A/J mice stained for perilipin-1 (red), laminin (green), and nuclei (DAPI). Rectus femoris muscle outlined in white (shows adipose accrual in the control-treated mice). **G.** Plot showing the quantification of total adipogenic area (labeled for perilipin-1) across entire quadriceps cross-section (top plot), and vastus region (bottom plot). Data indicate mean $\pm$ SEM. All * $p<.05$ vs. control-AAV via independent samples t-test. H&E scale bars (whole section images – 500 m, inlayed images – 100 m).

Supplemental Table

TableS1.	Sex (M,F)	Age at Injection (days)	Body Mass (g)
hASM-AAV	4,1	70 $\pm$ 2	28.62 $\pm$ 2.11
Control-AAV	2,3	70 $\pm$ 3	28.12 $\pm$ 2.09

Table S1. Baseline Mice Characteristics
Mouse gender composition, age, and body mass at the time of AA

Supplemental Methods

Sample Size Determination: We had also used the Vamorolone trial to assess the sample size for other measures used in this study namely, grip strength, histopathology, and eccentric muscle damaging assays. Grip strength measures from our Vamorolone studies demonstrated an effect size of 1.1 to detect improved hindlimb grip strength in BLAJ mice and effect size of 0.61 for forelimb grip strength, which translates to a required sample size of 4 mice per group to achieve statistical significance, assuming a two-tailed alpha set at 0.05, and power at 80%. Power calculation for eccentric contraction-induced injury showed an effect size of 0.624 with Vamorolone treatment, which identified 6 mice per treatment group to detect a significant effect of treatment on ex-vivo membrane stability upon mechanical eccentric injury. Lastly, histological measures of myofiber degeneration (IGM+ fibers in BLA/J mice) showed an effect size of 0.753, which requires 7 mice per group to observe statistical significance, assuming two-tailed alpha set at 0.05, and power at 80%.

Upon compiling this prior data from studies examining the effects of compounds or drugs that modify cell membrane lipids in LGMD2B (BLA/J mice), as hASM does, we ascertained that we would require 5 mice per group for our primary endpoint measure (membrane repair capacity) and 4-7 mice per treatment group to find statistically significant differences for additional end points tested. With the ethical and institutional mandate to minimize animal usage we chose to proceed with a sample size of 5 animals/group for this trial. Based on the reviewer’s suggestion to increase the group size we have consulted with our institutional biostatistician Dr. Heather Gordish-Dressman, who confirmed the robustness of our analysis and the calculation of the group size. She also independently advised that the marginal increase in significance that can be achieved by larger group size offers insufficient justification for such an increase.

Supplemental Videos

SV1. Caveolin Mobility (untreated cell)

Timelapse video of Caveolin-1 RFP-transfected C2C12 myoblast. Images acquired via spinning-disc confocal microscopy (60X/1.45NA oil objective), using a confocal diode laser of 560 nm, at the membrane-coverslip interface. Cells were imaged at 1-s intervals for a period of 3 minutes in CIM (Untreated). Video played at 10 frames/second.

SV2. Caveolin Mobility (hASM-treated cell)

Timelapse video of Caveolin-1 RFP-transfected C2C12 myoblast. Images were acquired via spinning-disc confocal microscopy (60X/1.45NA oil objective), using a confocal diode laser of 560 nm, at the membrane-coverslip interface. Cells were imaged at 1-s intervals for a period of 3 minutes in CIM supplemented with 6U/L hASM (hASM-treated – added after 1-minute of caveolin tracking). Video played at 10 frames/second. hASM addition is denoted at the time of addition in yellow text (upper left of the video panel).

SV3. GPI-GFP Membrane Endocytosis (untreated cell)

Timelapse video of GPI-GFP-transfected C2C12 myoblast. Images were acquired via spinning-disc confocal microscopy (60X/1.45NA oil objective), using a confocal diode laser of 488 nm, at mid-cell body z-plane. Cells were imaged at 1-minute intervals for a period of 20 minutes in CIM. Video played at 5 frames/second.

SV4. GPI-GFP Membrane Endocytosis (hASM-treated cell)

Timelapse video of GPI-GFP-transfected C2C12 myoblast. Images were acquired via spinning-disc confocal microscopy (60X/1.45NA oil objective), using a confocal diode laser of 488 nm, at mid-cell body z-plane. Cells were imaged at 1-minute intervals for a period of 20 minutes in CIM supplemented with 6U/L hASM (hASM-treated – added after 1-minute of caveolin tracking). Video played at 5 frames/second. hASM addition is denoted at the time of addition in yellow text (upper left of the video panel).

REFERENCES

1. Otto GP, Rathkolb B, Oestereicher MA, Lengger CJ, Moerth C, Micklich K, et al. Clinical Chemistry Reference Intervals for C57BL/6J, C57BL/6N, and C3HeB/FeJ Mice (Mus musculus). *J Am Assoc Lab Anim Sci.* 2016;55(4):375-86.
